# Entropy-Modulated Oxide–Metal Catalyst Architectures for Direct Ammonia Protonic Ceramic Fuel Cells

**DOI:** 10.1007/s40820-026-02194-9

**Published:** 2026-04-17

**Authors:** Dongyeon Kim, Dong Jae Park, Incheol Jeong, Seeun Oh, Hyeonggeun Kim, Mincheol Lee, Sang Won Lee, Kangyong Lee, Daehan Chung, Ki-Min Roh, Joongmyeon Bae, Tae Ho Shin, Kang Taek Lee

**Affiliations:** 1 KAIST InnoCORE PRISM-AI Center, KAIST, Daejeon, Republic of Korea; 2 Hydrogen Energy Materials Center, Korea Institute of Ceramic Engineering and Technology (KICET), Jinju-Si, Gyeongsangnam-Do Republic of Korea; 3 Resources Utilization Research Center, Korea Institute of Geoscience and Mineral Resources (KIGAM), Daejeon, Republic of Korea; 4 Department of Mechanical Engineering, KAIST, Daejeon, Republic of Korea; 5 KAIST Graduate School of Green Growth & Sustainability, Daejeon, Republic of Korea

**Keywords:** Protonic ceramic fuel cells (PCFCs), Ammonia, High-entropy perovskite, Anode catalyst layer, Density functional theory

## Abstract

Protonic ceramic fuel cells (PCFCs) operating on NH_3_ present a promising carbon-free energy pathway, yet their performance is often constrained by limited catalytic activity and degradation of conventional Ni-based anodes. Here, we report a high-entropy perovskite catalyst, Sr_2_Fe_1_Mo_0.2_Mn_0.2_Cr_0.2_Cu_0.2_Ni_0.2_O_6-*δ*_ (SFMMCCN), employed as an anode catalyst layer in direct ammonia-fed PCFCs. Upon reduction, SFMMCCN undergoes in situ exsolution of Ni–Fe–Cu alloy nanoparticles within a stable oxide matrix. This architecture provides synergistic enhancement of NH_3_ adsorption and decomposition through the combined effects of abundant surface acid sites and catalytically active alloy interfaces. As a result, the SFMMCCN cell achieves a record peak power density of 2.04 W cm⁻^2^ at 700 °C and demonstrates excellent operational stability for over 255 h at 600 °C under NH_3_ fuel. Compared to a bare cell, it exhibits significantly reduced polarization resistance and effectively suppresses Ni coarsening. Density functional theory calculations reveal that the high-entropy oxide framework, together with the exsolved Ni–Fe–Cu alloy, lowers the energy barriers for NH_3_ decomposition, thereby accelerating overall catalytic kinetics. These findings highlight entropy-controlled oxide–metal architectures as a powerful strategy to achieve both high performance and durability in NH_3_-fueled electrochemical systems, offering a viable pathway toward scalable and efficient hydrogen-based power generation.

**Supplementary Information:**

The online version contains supplementary material available at 10.1007/s40820-026-02194-9.

## Highlights

- Entropy-modulated oxide–metal catalyst exsolving Ni–Fe–Cu alloy nanoparticles from a high-entropy perovskite matrix enables efficient and durable ammonia decomposition.
- Density functional theory calculations reveal that the high-entropy oxide framework facilitates cation exsolution and lowers the kinetic barriers for NH_3_ decomposition; additionally, the exsolved Ni–Fe–Cu alloy nanoparticles exhibit markedly higher catalytic activity than single-metal surfaces.
- Direct ammonia protonic ceramic fuel cells (DA-PCFCs) incorporating the Sr_2_Fe_1_Mo_0.2_Mn_0.2_Cr_0.2_Cu_0.2_Ni_0.2_O_6-*δ*_ (SFMMCCN) catalyst layer achieve a record-high power density of 2.04 W cm^−2^ at 700 °C with stable operation for over 255 h under NH_3_ fuel, demonstrating the effectiveness of the entropy-modulated catalyst in designing durable and high-performance DA-PCFCs for carbon-free ammonia-to-power technologies.

**Supplementary Information:**

The online version contains supplementary material available at 10.1007/s40820-026-02194-9.

## Graphical abstract

**Figure:**
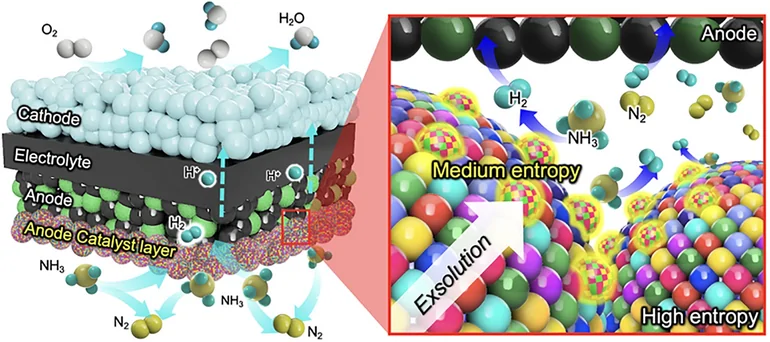

## Introduction

The global transition toward decarbonization and reduced reliance on fossil fuel intensified interest in hydrogen as a clean and versatile energy carrier [1]. Within this context, protonic ceramic fuel cells (PCFCs) have emerged as promising electrochemical devices that directly convert hydrogen into electricity through proton conduction, offering high efficiency and environmentally benign operation [2]. Despite these advantages, the large-scale adoption of hydrogen faces substantial challenges, including energy-intensive production pathways and limited distribution infrastructure [3]. Common delivery strategies—such as compression, liquefaction, or chemical storage in hydrides and carbonaceous compounds—remain technologically complex and economically unattractive [4].

Beyond hydrogen, carbon-based fuels such as methane and natural gas can be supplied to fuel cells, benefiting from fast kinetics and well-established distribution networks [5]. However, their utilization inevitably generates CO_2_ and other pollutants, which conflict with increasingly stringent environmental regulations. Ammonia (NH_3_) has therefore attracted considerable attention as an alternative energy carrier, due to its carbon-free composition, hydrogen-rich nature, favorable energy density, low flammability, and compatibility with existing large-scale storage and transport infrastructure [6, 7]. Leveraging these properties, direct ammonia-fed PCFCs (DA-PCFCs) operating at intermediate temperatures provide notable advantages, including higher system efficiency and reduced balance-of-plant complexity. Unlike conventional solid oxide fuel cells (SOFCs), which rely on oxygen-ion transport [8], the proton conduction mechanism of PCFCs inherently suppresses NO_x_ formation during ammonia utilization [9]. This synergy highlights DA-PCFCs as a particularly attractive pathway for clean and scalable power generation [10]. Nevertheless, the practical realization of DA-PCFCs is hindered by inadequate catalytic activity of conventional anodes toward NH_3_ decomposition. Ni-based cermet anodes, widely employed in PCFCs, show poor intrinsic activity, leading to incomplete conversion of NH_3_ to N_2_ and H_2_. This results in nitridation of metallic Ni to nickel nitride (Ni_3_N) via the reaction: NH_3_ + 3Ni → Ni_3_N + 1.5H_2_ [11]. Because Ni_3_N is unstable in H_2_-rich atmospheres, it readily reverts to Ni [12], causing cyclic phase transformations that induce structural degradation within the anode framework [13]. Over time, these processes undermine both catalytic efficiency and electrochemical stability, posing a critical obstacle to the reliable operation of DA-PCFC systems [14].

To overcome these limitations, surface engineering approaches have been explored to improve NH_3_ decomposition kinetics. One strategy involves generating catalytically active nanoparticles on the anode surface. For instance, Liu et al*.* developed a Ni-Ba(Zr_0.1_Ce_0.7_Y_0.1_Yb_0.1_)_0.94_Ru_0.03_Fe_0.03_O_3-*δ*_ (BZCYYbRF) anode, where Ru and Fe co-doping facilitated the exsolution of RuFe alloy nanoparticles under reducing conditions, yielding a peak power density of 807 mW cm^−2^ at 650 °C with NH_3_ fuel [15]. Similarly, Shao et al*.* reported a Ni-Ba(Zr_0.1_Ce_0.7_Y_0.1_Yb_0.1_)_0.95_Pd_0.05_O_3-*δ*_ (BZCYYbPd) anode, in which Pd incorporation enhanced ammonia decomposition activity and improved overall performance [16]. Infiltration-based methods have also been pursued; for example, Ru_0.95_Cu_0.05_Ni_x_ (RCN) nanoparticles introduced via one-step infiltration improved long-term stability, reducing the voltage degradation rate to 0.016 V over 100 h, compared to 0.095 V in the pristine anode [17]. Nevertheless, the reliance on noble metals such as Ru and Pd raises cost concerns [18], and challenges including direct NH_3_–Ni interactions and Ni particle agglomeration persist [10].

Recent studies have demonstrated that interface engineering is an effective route to improving the activity and durability of non-noble metal catalysts for ammonia decomposition. Engineered metal–oxide interfaces, in turn, regulate surface reaction energetics and stabilize active sites under harsh NH_3_ environments [9, 19]. Alternatively, beyond tailoring local interfacial chemistry, the incorporation of an anode catalyst layer (ACL) has been proposed as a complementary strategy to spatially decouple NH_3_ decomposition from the Ni-based anode, thereby minimizing direct Ni–NH_3_ contact and enhancing chemical stability [20, 21]. Pan et al*.* demonstrated tubular PCFCs employing a catalytic Fe layer, which suppressed Ni_3_N formation and improved durability. Density functional theory (DFT) calculations further revealed that Fe promotes NH_3_ decomposition due to its favorable nitrogen adsorption energetics [22]. More recently, Mo-containing perovskite oxides such as Sr_2_Fe_2-x_Mo_x_O_6-*δ*_ (SFM) have emerged as attractive ACL candidates, as Mo-induced acidic sites accelerate NH_3_ decomposition [9, 23]. He et al*.* reported that a Sr_2_Fe_1.35_Mo_0.45_Cu_0.2_O_6-*δ*_ (SFMC) layer, integrated atop a Ni-BZCYYb anode, delivered excellent performance above 650 °C. Under reducing conditions, exsolved Fe and Cu nanoparticles alloyed with Ni, producing highly active Ni–Cu and Ni–Fe species that markedly improved NH_3_ conversion [24]. Building on these findings, we hypothesized that co-doping SFM with both acidic and reducible elements could synergistically maximize catalytic efficiency for NH_3_ decomposition. Acidic dopants more potent than Mo may promote N–H bond cleavage, while reducible elements with strong exsolution tendencies can generate highly active metallic species under operating conditions. Furthermore, adopting a high-entropy oxide design—incorporating five or more equimolar cations—may introduce entropy-stabilization effects, thereby improving long-term durability under fuel cell conditions [25, 26].

In this study, we designed and synthesized a high-entropy perovskite oxide catalyst, Sr_2_Fe_1_Mo_0.2_Mn_0.2_Cr_0.2_Cu_0.2_Ni_0.2_O_6-*δ*_ (SFMMCCN), incorporating Mn and Cr as acidic dopants and Cu and Ni as reducible dopants. SFMMCCN was systematically evaluated as an ACL for DA-PCFCs. Structural integrity and entropy-stabilization effects were confirmed through comprehensive physicochemical characterization, while catalytic activity and durability toward NH_3_ decomposition were assessed under relevant fuel conditions. Complementary DFT calculations provided mechanistic insights into the role of entropy-driven design in promoting NH_3_ decomposition kinetics. Finally, a full DA-PCFC incorporating SFMMCCN as the ACL was fabricated, demonstrating strong functional viability in practical cell operation.

## Experimental Section

### Material Preparation

Powders of Sr_2_Fe_1.5_Mo_0.5_O_6-*δ*_ (SFM), Sr_2_Fe_1_Mo_0.6_Cu_0.2_Ni_0.2_O_6-*δ*_ (SFMCN), and SFMMCCN were synthesized via a sol–gel process. For SFM, high-purity Sr(NO_3_)_2_, ${\mathrm{Fe}}({\mathrm{NO}}_{3})_{3}\cdot{9{\mathrm{H}}}_2{\mathrm{O}}$, and (NH_4_)_2_MoO_4_ were used as cation precursors. For SFMMCCN, the same precursors were combined with Mn(NO_3_)_2_·6H_2_O, Cr(NO_3_)_3_·9H_2_O, Cu(NO_3_)_2_·3H_2_O, and Ni(NO_3_)_2_·6H_2_O in the appropriate molar ratios. All precursors were dissolved in deionized water at 80 °C, after which ethylenediaminetetraacetic acid (EDTA) and citric acid were introduced. The solution pH was adjusted to 8 by the controlled addition of ammonia. The gel formed upon evaporation was heated at 300 °C for 2 h, and the resulting precursor was calcined at 1000 °C for 5 h to obtain a phase-pure perovskite. The powders were subsequently ball-milled in ethanol to produce fine particles. The BaSc_0.1_Ta_0.1_Co_0.8_O_3-*δ*_ (BSTC) cathode was synthesized by a solid-state reaction using stoichiometric precursor compositions [27].

### Physicochemical Characterizations

High-resolution X-ray diffraction (XRD) was performed using Cu-K*α*_1_ radiation and a Ge (111) monochromator over a 2θ range of 20°–80°. The microstructure of SFMMCCN pellets and single cells was examined by scanning electron microscopy (SEM) with energy-dispersive X-ray spectroscopy (EDS) (JEOL, JSM-IT800). Atomic-scale features were further investigated using a high-resolution transmission electron microscope (HR-TEM; Thermo Fisher, Spectra Ultra). X-ray photoelectron spectroscopy (XPS; K-Alpha, Thermo VG Scientific) with monochromatic Al K*α* radiation was employed to determine the valence states of the elements. NH_3_ temperature-programmed desorption (NH_3_-TPD) was performed on samples pretreated at 500 °C in He for 2 h, reduced at 700 °C in H_2_ for 2 h, and then exposed to NH_3_. Desorption was monitored during heating at 10 °C min⁻^1^ using an AutoChem II 2920 (Micromeritics).

### Catalytic Activity Test

The catalytic activities of SFM, SFMMCCN, and Ni-BaZr_0.4_Ce_0.4_Y_0.1_Yb_0.1_O_3-*δ*_ (BZCYYb) were evaluated in a fixed-bed quartz reactor. Each catalyst powder (0.2 g) was pre-reduced in a flow of H_2_ (50 sccm) at 700 °C for 2 h, followed by the introduction of NH_3_ (20 sccm). The specific surface areas of all catalysts were determined by Brunauer–Emmett–Teller (BET) N_2_ adsorption measurements (Fig. S1). The NH_3_ decomposition ratio was measured as a function of temperature. Residual NH_3_ and H_2_O in the effluent were removed using dilute H_2_SO_4_ solution and CaSO_4_ absorbent, respectively. Effluent gas composition was measured with a mass flow meter (Bronkhorst, Ruurlo, Netherlands). The NH_3_ conversion efficiency, corresponding to a theoretical product ratio of 75% H_2_ and 25% N_2_, was determined by the following Eq. (1)

1 $${\mathrm{NH}}_{3} \; \mathrm{conversion} \; (\%)=\frac{{F}_{\mathrm{out}}}{2{F}_{\mathrm{in}}}\times 100$$

where *F*_in_ and *F*_out_ denote the inlet and outlet gas flow rates, respectively [28].

### Computational Details

The DFT calculations were conducted with the Vienna ab initio simulation package (VASP) [29]. The Perdew–Burke–Ernzerhof generalized gradient approximation (GGA-PBE) was considered for the exchange–correlation functionals. Valence configurations were 4*p*^6^3*d*^6^4*s*^1^ for Mn, 4*p*^6^3*d*^5^4*s*^1^ for Cr, 3*d*^10^4*s*^1^ for Cu, 3*d*^9^4*s*^1^ for Ni, 4*s*^2^4*p*^6^4*d*^5^5*s*^1^ for Mo, 4*s*^2^4*p*^6^5*s*^2^ for Sr, 3*d*^6^4*s*^1^ for Fe, and 2*s*^2^*p*^4^ for O. DFT + U approach was applied and the values employed for Mn, Cr, Cu, Ni, Mo, and Fe were 3.9, 3.7, 4.0, 6.2, 4.38, and 5.3 eV, respectively. Plane waves with an energy cutoff of 450 eV were used. A Monkhorst–Pack [30] *k*-point mesh of 1 × 2 × 1 and 3 × 3 × 1 was applied to the 156-atom perovskite and 54-atom metal (Ni and Ni–Fe–Cu) slabs, respectively. The convergence threshold for electronic self-consistent iterations was 10^−6^ eV cell^−1^. Cell parameters and atomic positions were relaxed until the remaining force was less than 1 × 10^−1^ eV Å^−1^. Each slab was separated along the *z*-axis by a 20 Å of vacuum region. NH_3_ adsorption sites were determined by identifying those with the lowest energy cost among all possible cation sites in perovskites and atop, bridge, FCC, and HCP sites in metals.

### Single Cell Fabrication

Anode-supported protonic ceramic fuel cells (PCFCs) were fabricated in a multilayer configuration consisting of a NiO–BZCYYb anode, a NiO–BZCYYb anode functional layer, a BZCYYb electrolyte, and a BSTC cathode. For the supporting layer slurry, NiO (Sumitomo) and BZCYYb powder (Kceracell) were blended at a 6:4 weight ratio, followed by sequential addition of ethanol and toluene as solvents, Hypermer KD-1 (CRODA) as a dispersant, polyvinyl butyral (Eastman Chemical Company) as a binder, di-n-butyl phthalate (Junsei) as a plasticizer, and poly(methyl methacrylate) (Sunjin Chemical) as a pore former. Slurries for the functional layer and electrolyte (prepared without pore former) were produced using the same procedure. The resulting slurries were tape-cast, dried, and laminated to form green tapes. These laminates were pre-sintered at 900 °C for 3 h to remove organic components then sintered at 1400 °C for 5 min in a microwave furnace (Unicera, UMF-04; 2.45 GHz, 2 kW). The SFMMCCN anode catalyst layer (ACL) ink was prepared by mixing SFMMCCN powder with a commercial binder system (ElectroScience, 441 ESL), which was brush-coated onto the anode surface and sintered at 950 °C for 3 min in a microwave furnace. The BSTC cathode slurry was then screen-printed onto the electrolyte and sintered at 850 °C for 3 min in a microwave furnace.

### Electrochemical Characterizations

Single cells were affixed to an alumina tube and sealed hermetically with Ceramabond 571 (Aremco). Prior to electrochemical testing of the DA-PCFCs, the anode was reduced in humidified H_2_ (3% H_2_O, 50 sccm) and subsequently exposed to NH_3_ (50 sccm), while the BSTC cathode was supplied with humidified air (3% H_2_O, 50 sccm). Current–voltage (I–V) curves and electrochemical impedance spectroscopy (EIS) were recorded using a potentiostat (Bio-Logic, VMP-300). EIS spectra were collected over a frequency range of 1 MHz–0.1 Hz with an AC perturbation amplitude of 50 mV.

## Results and Discussion

### Physicochemical Characterization of the Materials

**Fig. 1:**
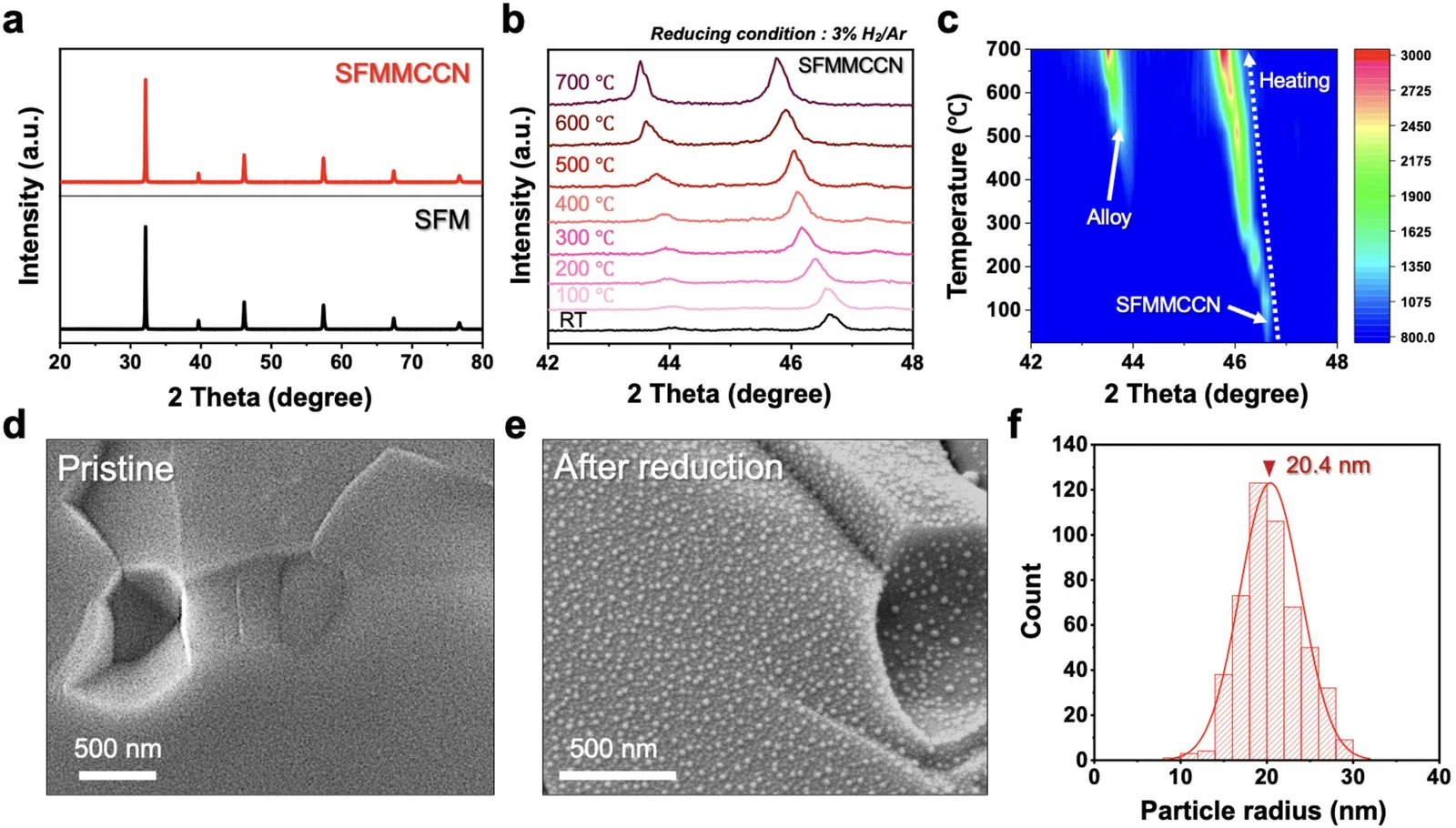
**a** XRD patterns of the as-synthesized SFM and SFMMCCN. In situ HT-XRD **b** patterns and **c** contour plot of SFMMCCN under reducing atmosphere (3% H_2_/Ar) from RT to 700 °C. SEM images of the SFMMCCN pellet surface in the **d** as-synthesized and **e** after reduction. **f** Particle size distribution histogram of exsolved nanoparticles on the surface of the reduced SFMMCCN pellet

SFM and SFMMCCN, were synthesized via a sol–gel method. Figure 1a presents the XRD patterns. The results confirm the formation of well-defined double perovskite structures in both materials, indicating successful incorporation of Mn, Cr, Cu, and Ni into the SFM lattice without secondary phases. To probe the structural evolution under reduction conditions, in situ high-temperature XRD (HT-XRD) was performed in 3% H_2_/Ar from room temperature (RT) to 700 °C. As shown in Fig. 1b, c, the lattice progressively expands with increasing temperature, reflecting the combined contributions of thermal expansion and reduction-induced chemical expansion [31]. Notably, a new diffraction feature emerges near 44° above 400 °C, consistent with the nucleation of a metallic phase via exsolution. In contrast, no additional diffraction peaks associated with exsolved metallic species were detected for SFM after reduction (Fig. S2). To validate these findings under PCFC operating conditions, SFMMCCN pellets were reduced at 700 °C in pure H_2_. The SEM images (Fig. 1d, e) reveal a pronounced surface transformation: the pristine surface evolves into one decorated with dense, uniformly distributed nanoparticles after reduction. This confirms the successful activation of exsolution under practical thermal and chemical conditions. Furthermore, particle size distribution analysis indicates an average nanoparticle radius of ~ 20.4 nm, underscoring both the high dispersion and nanoscale uniformity of the exsolved phase (Fig. 1f).

**Fig. 2:**
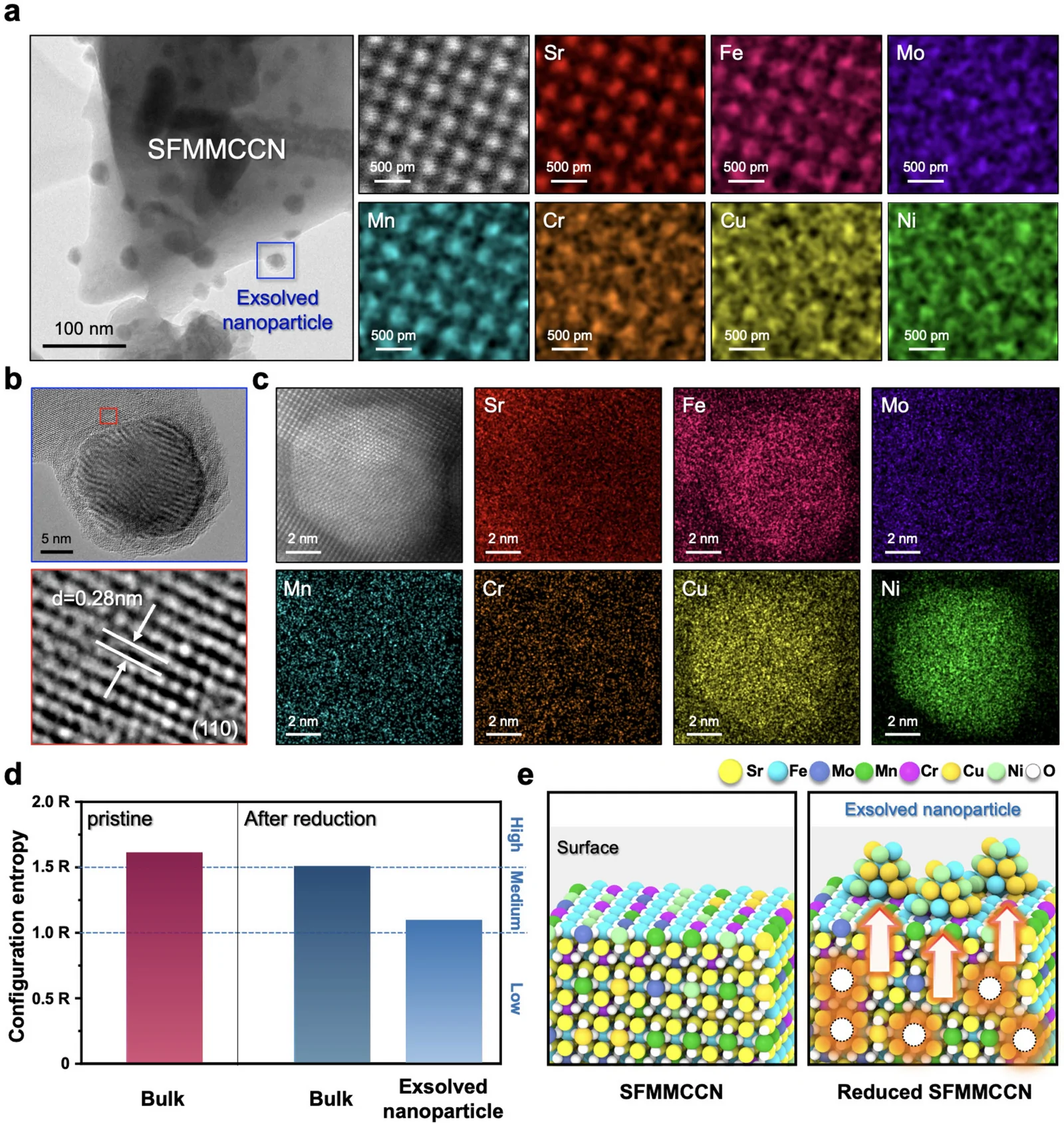
**a** HR-TEM, HAADF-STEM and elemental mapping images of reduced SFMMCCN powder. **b** HR-TEM, lattice fringe, and **c** HAADF-STEM and EDS mapping images of exsolved nanoparticles. **d** Configuration entropy of the pristine and reduced SFMMCCN. **e** Schematic illustration of the in situ entropy-controlled process

Figure 2a displays the HR-TEM image of reduced SFMMCCN, revealing a well-defined perovskite lattice with distinct sublattices occupied by multiple transition-metal cations. After reduction, finely dispersed nanoparticles were observed within the bulk matrix. High-angle annular dark-field scanning transmission electron microscopy (HAADF-STEM) combined with the EDS confirms a uniform elemental distribution across the reduced perovskite oxide, validating the successful incorporation of Mn, Cr, Cu, and Ni into the B-site lattice. Figure 2b shows the HR-TEM lattice fringe of an exsolved nanoparticle. The measured *d*-spacing of 0.28 nm corresponds to the (110) plane of the perovskite phase, indicating coherent lattice integration between the nanoparticle and the host matrix. The nanoparticle exhibited an average diameter of ~ 20 nm, with robust particle–matrix interfaces that mitigate detachment and aggregation—two key degradation pathways that typically compromise long-term catalytic performance [32, 33]. Figure 2c further provides EDS elemental maps of the exsolved nanoparticles, showing homogeneous distributions of Ni, Fe, and Cu, consistent with the formation of a Ni–Fe–Cu alloy. Additional structural confirmation is presented in Fig. S3, where HR-TEM and fast Fourier transform (FFT) analysis suggest that the exsolved phase adopts a face-centered cubic (FCC) structure, in agreement with reported Ni-based alloys [34, 35].

To characterize the atomic distribution and calculate configurational entropy (Δ S_config_) of both the exsolved alloy and the bulk perovskite, STEM-EDS analysis was performed (Fig. S4–S6). The ${\Delta \mathrm{S}}_{\mathrm{config}}$ of metallic alloys is calculated using Eq. (2) [36]:

2 $$\Delta S_{{{\mathrm{config}}}} = - R\sum\limits_{{{\mathrm{i}} = 1}}^{{\mathrm{N}}} {x_{{\mathrm{i}}} lnx_{{\mathrm{i}}} }$$

where *R* is the gas constant, *N* denotes the number of constituent elements, and *x*_i_ is the mole fraction of the *i*-th component. Based on this metric, alloys are classified as high-, medium-, or low-entropy systems when Δ S_config_ ≥ 1.5R, 1.0R $\le \Delta$ S_config_ ≤ 1.5R, and Δ S_config_ ≤ 1.0R, respectively [37]. For perovskite oxides, ${\Delta \mathrm{S}}_{\mathrm{config}}$ can similarly be expressed as Eq. (3) [38]:

3 $$\Delta S_{{{\mathrm{config}}}} = - R\left[ {\left( {\sum\limits_{a = 1}^{{\mathrm{A}}} {x_{{\mathrm{a}}} lnx_{a} } + \sum\limits_{b = 1}^{{\mathrm{B}}} {x_{{\mathrm{b}}} lnx_{b} } } \right)_{{{\mathrm{cation}}}} + \left( {\sum\limits_{c = 1}^{{\mathrm{C}}} {x_{c} lnx_{c} } } \right)_{{{\mathrm{anion}}}} } \right]$$

where *A* and *B* denote the number of species occupying the A- and B-sites, respectively, *C* the number of anion types, and *x*_a_, *x*_b_, and *x*_c_ the mole fractions. As shown in Fig. 2d, pristine SFMMCCN exhibits a configurational entropy of 1.60 R, qualifying as a high-entropy perovskite oxide (HEPO). Upon reduction, the bulk retains a high-entropy state, albeit slightly lower at 1.50 R. In contrast, the exsolved Ni–Fe–Cu alloy displays a medium-entropy value of 1.09 R. The schematic in Fig. 2e illustrates this entropy-controlled process: under reducing conditions, selective exsolution of a medium-entropy alloy occurs from the high-entropy oxide matrix.

**Fig. 3:**
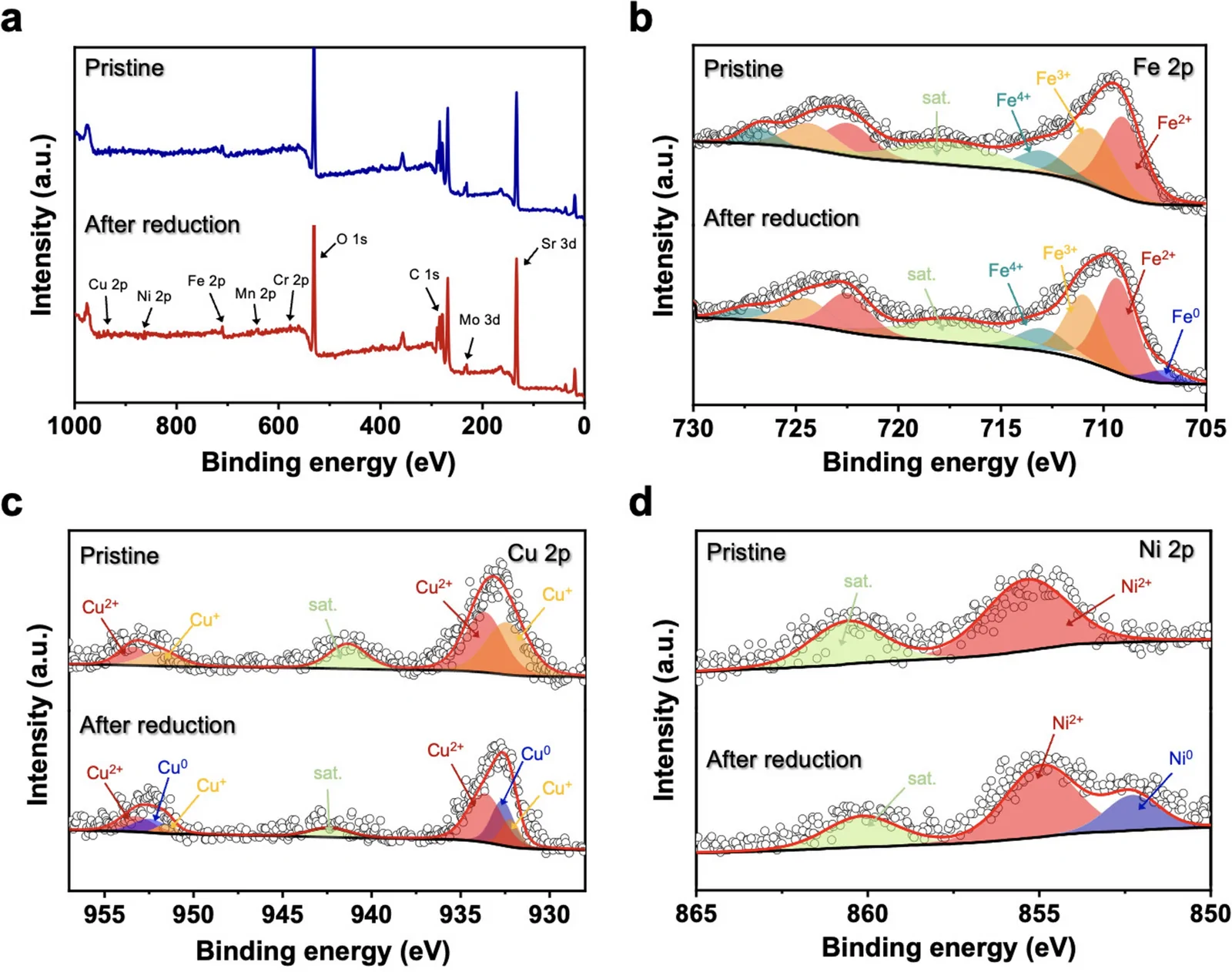
XPS spectra of **a** survey, **b** Fe 2*p*, **c** Cu 2*p*, and **d** Ni 2*p* for SFMMCCN before and after reduction at 700 °C for 2 h in a 100% H_2_ atmosphere

The XPS analysis was employed to investigate the valence states of the constituent elements in SFMMCCN before and after exposure to reducing conditions. All spectra were calibrated using the C 1*s* as a reference. As shown in Fig. 3a, characteristic binding energy signals corresponding to Sr, Fe, Mo, Mn, Cr, Cu, and Ni were consistently observed in both the pristine and reduced samples. Detailed spectral deconvolution of the Fe 2*p*, Cu 2*p*, and Ni 2*p* regions is presented in Fig. 3b–d. In the pristine sample, only oxidized states of the transition metals were identified. After reduction at 700 °C for 2 h in a pure H_2_ atmosphere, additional features indicative of metallic species emerged. Specifically, a distinct Fe^0^ peak appears at ~ 707 eV in the Fe 2*p* spectrum (Fig. 3b) [10]. The corresponding Fe 2*p* fitting parameters are summarized in Table S1. In addition, the Cu 2*p* spectra (Fig. 3c) revealed characteristic Cu^0^ peaks near 933 and 953 eV [39]. Figure 3d displays the Ni 2*p* spectrum, where a peak at ~ 852 eV is assigned to metallic Ni^0^ species [17]. Complementary XPS analyses of Mo, Mn, Cr, and O species before and after reduction are presented in Fig. S7. Collectively, these observations confirm the reduction of Ni, Fe, and Cu, leading to the in situ exsolution of a Ni–Fe–Cu alloy from the SFMMCCN perovskite matrix, consistent with the structural evidence in Fig. 2. The formation of this multi-metallic alloy is anticipated to significantly improve catalytic activity for ammonia decomposition through synergistic effects and enhanced surface reactivity compared to monometallic catalysts. A detailed evaluation of this catalytic performance is provided in the following section.

### Catalytic Properties for NH_3_ Decomposition

**Fig. 4:**
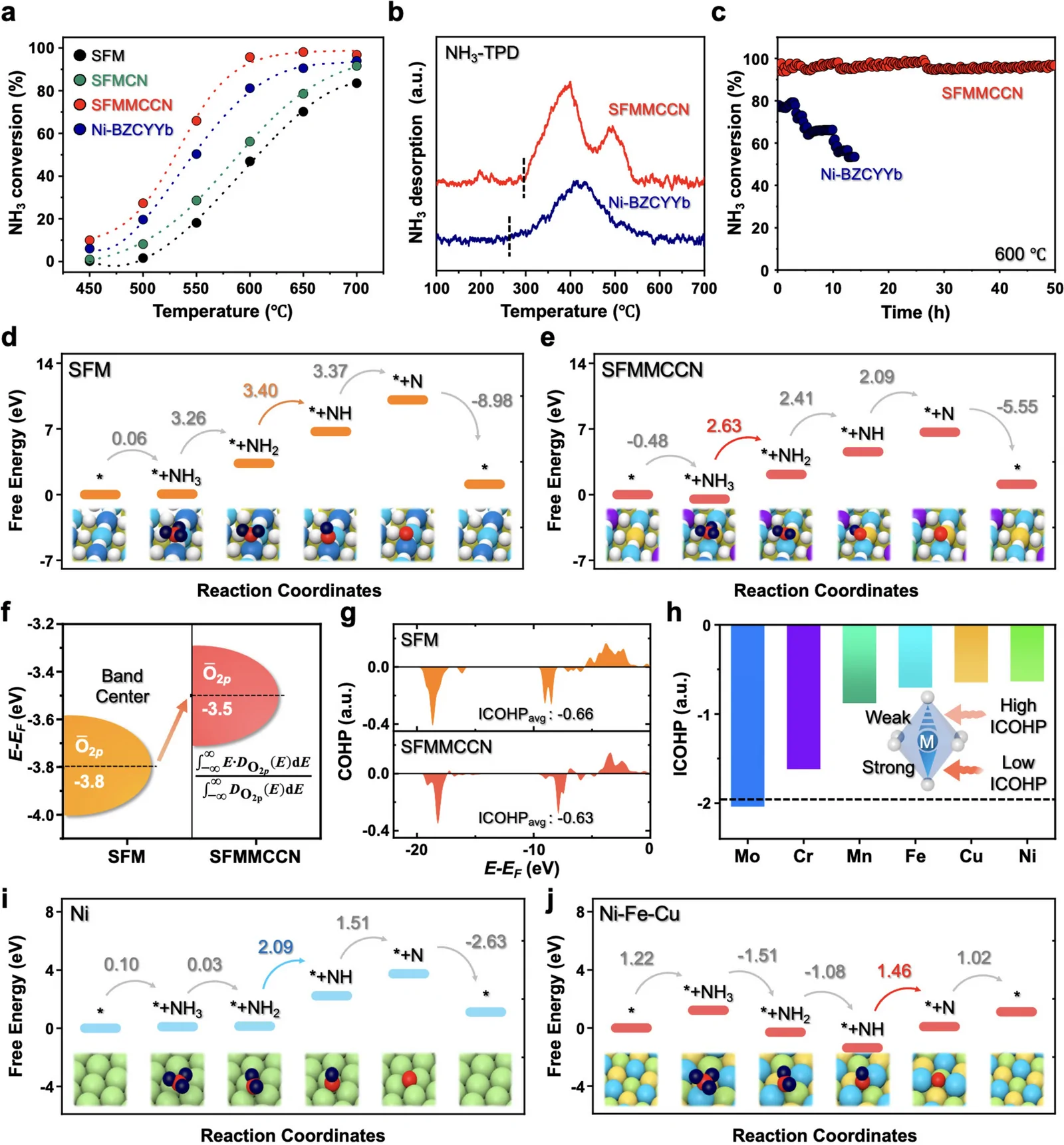
**a** NH_3_ conversion of SFM, SFMCN, SFMMCCN, and Ni-BZCYYb measured across the temperature range of 450–700 °C. **b** NH_3_-TPD profiles and **c** durability test of SFMMCCN and Ni-BZCYYb. Energy profiles for NH_3_ decomposition on **d** SFM and **e** SFMMCCN. **f** O *2p* band center. **g** COHP profiles and average ICOHP values of cation–oxygen pairs. **h** ICOHP of high-entropy elements in SFMMCCN. O *2p* band center. Energy profiles for NH_3_ on **i** Ni and **j** Ni–Fe–Cu alloy

To assess the catalytic potential of SFMMCCN as an ACL in DA-PCFCs, its NH_3_ conversion efficiency was compared against those of a low-entropy perovskite catalyst (SFM), a medium-entropy perovskite catalyst (SFMCN), and a bare anode (Ni-BZCYYb). Prior to testing, all materials were thermally treated at 700 °C for 2 h under a pure H_2_ atmosphere, identical to the conditions used during cell operation. As shown in Fig. 4a, SFMMCCN exhibits a remarkable NH_3_ conversion of 96% at 600 °C, far exceeding that of SFM (47%) and SFMCN (57%). Interestingly, the bare Ni-BZCYYb anode exhibits higher NH_3_ conversion than SFM and SFMCN, reflecting the intrinsically high catalytic activity of metallic Ni toward NH_3_ decomposition. These observations imply that the exceptional reactivity of SFMMCCN arises from the combined effects of high configurational entropy and in situ formation of Ni–Fe–Cu alloy nanoparticles. Since surface acidity is a critical factor influencing NH_3_ adsorption and subsequent decomposition, the NH_3_-TPD was performed. As depicted in Fig. 4b, SFMMCCN exhibited a higher onset temperature compared to Ni-BZCYYb, signifying the presence of stronger acid sites favorable for NH_3_ adsorption. Materials with stronger NH_3_ adsorption generally facilitate more efficient surface reactions, thereby accelerating decomposition kinetics [40]. To exclude the possible contribution of exsolved alloy nanoparticles to the NH_3_-TPD response, additional control experiments were performed without prior H_2_ reduction pretreatment, thereby minimizing alloy exsolution before the measurement (Figs. S8 and S9). Under these exsolution-suppressed conditions, SFMMCCN still exhibits an elevated NH_3_ desorption onset compared with Ni-BZCYYb, indicating that the enhanced NH_3_ adsorption is not governed by metallic nanoparticles. Moreover, comparison with the Mn- and Cr-free control sample (SFMCN) further confirms that the strengthened surface acidity originates from the intrinsic oxide matrix induced by Mn and Cr incorporation. The long-term catalytic durability is shown in Fig. 4c. While SFMMCCN sustained nearly complete NH_3_ conversion for over 50 h at 600 °C, Ni-BZCYYb exhibited rapid deactivation within 20 h. This degradation is attributed to Ni nanoparticle coarsening, triggered by repeated phase nitridation (Ni → Ni_3_N) and subsequent reversion to metallic Ni. Consistent with this mechanism, SEM analysis after durability testing (Fig. S10) revealed pronounced structural degradation of Ni-BZCYYb, whereas SFMMCCN preserved its morphology. Post-test XRD and STEM–EDS analyses further indicate that beyond the structural evolution of Ni, the Ni-BZCYYb undergoes phase separation of the BZCYYb component under NH_3_ operation, accompanied by BaO formation (Figs. S11 and S12). In contrast, the SFMMCCN remains structurally and chemically intact after long-term operation, with no detectable secondary phases observed, indicating excellent phase stability (Figs. S13 and S14). XPS analysis further confirms that the chemical states of the exsolved Ni–Fe–Cu alloy nanoparticles are largely preserved after durability testing (Fig. S15).

### DFT Analysis of NH_3_ Decomposition and Exsolution Mechanism

Figure 4d and e display DFT calculation results to determine energy profiles for NH_3_ decomposition on SFM and SFMMCCN, respectively. The reaction pathway consists of 5 elementary steps including the adsorption of NH_3_ (Eq. 4), a series of deprotonation generating hydrogen gas (Eqs. 5–7), and desorption of nitrogen (Eq. 8) as follows:

4 $$^{*} \, + {\text{ NH}}_{{3}} \left( {\mathrm{g}} \right) \, \to \, ^{*}{\mathrm{NH}}_{{3}}$$

5 $$^{*}{\mathrm{NH}}_{{3}} \to \, ^{*}{\mathrm{NH}}_{{2}} + \frac{{1}}{{2}}{\mathrm{H}}_{{2}} \left( {\mathrm{g}} \right)$$

6 $$^{*} {\mathrm{NH}}_{{2}} \to \, ^{*} {\text{NH }} + \frac{{1}}{{2}}{\mathrm{H}}_{{2}} \left( {\mathrm{g}} \right)$$

7 $$^{*}{\text{NH }} \to \, ^{*}{\text{N }} + \frac{{1}}{{2}}{\mathrm{H}}_{{2}} \left( {\mathrm{g}} \right)$$

8 $$^{*}{\text{N }} \to \, ^{*} \, + \frac{{1}}{{2}}{\mathrm{N}}_{{2}} \left( {\mathrm{g}} \right)$$

Optimized structure models for SFM, SFMMCCN, Ni, and Ni–Fe–Cu systems are provided in Figs. S16–S19, respectively. The adsorption site * was determined by searching the configuration with the lowest energy cost. The reaction sites considered in this study, including atop, bridge, FCC, and HCP sites, along with their corresponding energetics for the SFM, SFMMCCN, Ni, and Ni–Fe–Cu models, are presented in Fig. S20. For the oxide systems (SFM and SFMMCCN), only metal atop sites were considered. In the Ni–Fe–Cu model, the adsorbate initially placed on the FCC site was found to relocate to the Fe atop site upon structural relaxation. The calculated highest energy barrier was 3.40 eV for SFM, compared to 2.63 eV for SFMMCCN, confirming that the high-entropy perovskite lowers the kinetic barriers for NH_3_ decomposition. We further investigated the driving force for exsolution in SFMMCCN. Figure 4f displays that the O 2*p* band center in SFMMCCN (− 3.5 eV) lies at a higher energy level than in SFM (− 3.8 eV), indicating that oxygen vacancy formation—and hence cation exsolution—is more favorable in SFMMCCN. Crystal orbital Hamilton population (COHP) analysis (Fig. 4g) revealed less negative integrated COHP (ICOHP) value for SFMMCCN compared with SFM, suggesting weaker cation–oxygen bonds and greater exsolution propensity. The ICOHP values for individual elements in SFMMCCN (Fig. 4h) further showed that Ni, Cu, and Fe possess the weakest bonding to oxygen, correlating with the experimentally observed exsolved alloy composition (Fig. 2). Finally, the energy profiles for NH_3_ decomposition were compared between bare Ni and the exsolved Ni–Fe–Cu alloy (Fig. 4i, j). The maximum energy barrier for Ni was 2.09 eV, while the exsolved Ni–Fe–Cu alloy exhibited a significantly lower barrier of 1.46 eV. The overall order of energy barriers was: Ni–Fe–Cu (1.46 eV) < Ni (2.09 eV) < SFMMCCN (2.63 eV) < SFM (3.40 eV).

These findings confirm that the exsolved metallic alloy provides superior catalytic activity in NH_3_ decomposition compared to either the parent. We suggest that transition-state searches could further provide detailed information on reaction kinetics, including energy barriers, diffusion processes, and N_2_ formation. To this end, developing computational methodologies that incorporate high-entropy configurations without compromising accuracy would be an interesting topic for future work.

### Electrochemical Performance

**Fig. 5:**
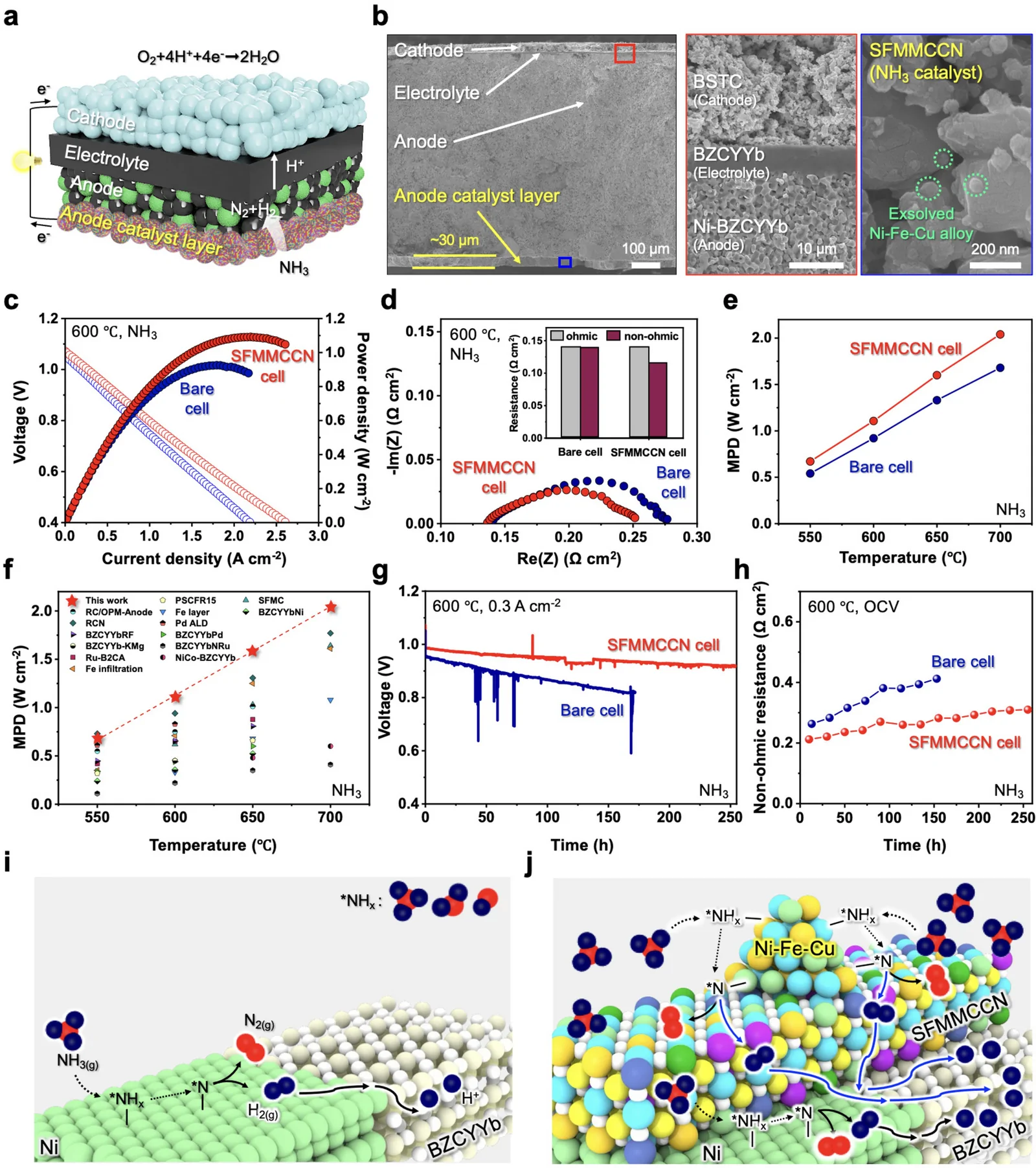
**a** Conceptual schematic and **b** cross-sectional SEM images of the DA-PCFC incorporating the SFMMCCN ACL. **c** *I–V–P* curves and **d** Nyquist plots of DA-PCFCs with ACL (SFMMCCN cell) and without ACL (bare cell) under NH_3_ fuel at 600 °C. **e** MPD comparison between SFMMCCN and bare cells over the 550–700 °C range. **f** Comparison of the MPDs of various DA-PCFCs. **g** Long-term durability test at 0.3 A cm^−2^ using NH_3_ fuel. **h** Comparison of non-ohmic resistance during long-term durability testing. Schematic representations of NH_3_ adsorption and stepwise decomposition pathways on Ni-BZCYYb anodes **i** without the ACL and **j** with the ACL

To assess the practical application of SFMMCCN as an ACL for DA-PCFCs, a cell configuration was constructed as depicted in Fig. 5a. In this system, NH_3_ is supplied directly to the anode, where it is initially adsorbed on the ACL and subsequently decomposed into N_2_ and H_2_ by the catalyst sites. The cross-sectional SEM image in Fig. 5b shows the assembled cell structure, comprising a SFMMCCN ACL, a Ni-BZCYYb anode, a BZCYYb electrolyte, and a BSTC cathode (hereafter referred to as the SFMMCCN cell). The ACL was deposited uniformly on the anode with a thickness of approximately 30 µm, forming a well-adhered interface without observable delamination. This robust interfacial contact can be attributed, in part, to the favorable thermo-mechanical compatibility between SFMMCCN and NiO–BZCYYb, as evidenced by their similar thermal expansion coefficients (TECs) measured by thermal dilatometry (Fig. S21). High-resolution imaging revealed well-dispersed Ni–Fe–Cu alloy nanoparticles exsolved from the SFMMCCN surface, with no signs of agglomeration. For comparison, a reference cell with an identical configuration but without the ACL was fabricated, hereafter denoted as the bare cell (Fig. S22). Figure 5c presents the electrochemical performance under NH_3_ fuel at 600 °C. The SFMMCCN cell achieved a maximum power density (MPD) of 1.11 W cm^−2^, reflecting a 32.1% enhancement relative to the bare cell. This improvement underscores the catalytic role of the SFMMCCN layer in facilitating NH_3_ decomposition. Impedance spectra collected at 600 °C (Fig. 5d) further support this conclusion: compared to the bare cell, the SFMMCCN cell exhibits markedly lower non-ohmic resistances, as summarized in the inset, suggesting more efficient catalytic conversion of NH_3_ into N_2_ and H_2_. Consistently, distribution of relaxation time (DRT) analysis (Fig. S23) reveals a substantially reduced high-frequency contribution for the SFMMCCN cell, reflecting accelerated anodic kinetics enabled by rapid NH_3_-to-H_2_ conversion and subsequent H_2_ → H^+^ electrochemical reactions. In contrast, a slightly increased low-frequency contribution is observed, which is attributed to the relatively lower porosity of the SFMMCCN ACL compared with the bare Ni-BZCYYb anode. Nevertheless, this modest gas-diffusion penalty is outweighed by the pronounced enhancement in anodic charge-transfer kinetics, resulting in superior overall performance under NH_3_ fuel. Figures S24 and S25 show temperature-dependent impedance spectra for both PCFCs. Figure 5e compares MPD values across the range of 550–700 °C, demonstrating that the SFMMCCN cell consistently outperformed the bare cell. The corresponding *I–V–P* curves are presented in Figs. S26 and S27. As summarized in Fig. 5f and Table 1, the SFMMCCN cell also surpasses the performance of other state-of-the-art DA-PCFCs. To the best of our knowledge, this is the first demonstration of a DA-PCFC achieving an MPD of 2.04 W cm^−2^ at 700 °C, surpassing all previously reported values irrespective of catalyst design.

**Table 1:** Comparison of the of various DA-PCFCs at the temperature range of 550–700 °C

Cathode	Electrolyte	Anode	Anode catalyst	MPD (W cm^−2^)				References
				Temperature (°C)				
				550	600	650	700	
BSTC	BZCYYb4411	Ni-BZCYYb4411	SFMMCCN layer	0.67	1.11	1.60	2.04	This work
BSTC	BZCYYb4411	Ni-BZCYYb4411	–	0.54	0.92	1.33	1.68	
BCFZYN^a^	BZCYYb1711^b^	Ni-BZCYYb1711	PSCFR15^c^ layer	0.32	0.45	0.63	–	[10]
PBSCF^d^	BZCYYb1711	Ni-BZCYYb1711	SFMC^e^ layer	–	0.62	1.03	1.64	[24]
PBSCF	BZCYYb1711	RC/OPM-Anode^f^	–	0.55	0.75	1.01	–	[41]
PBSCF	BZCYYb1711	Ni-BZCYYb1711	Fe layer	–	0.33	0.69	1.08	[22]
BCCY^g^	BZCYYbN^h^	Ni-BZCYYbN	–	0.24	0.36	0.52	–	[42]
PBSCF	BZCYYb1711	RCN^i^-decorated Ni-BZCYYb1711	–	0.73	0.94	1.31	1.77	[17]
PBSCF	BZCYYb2611^j^	Pd-decorated Ni-BZCYYb2611	–	0.34	0.61	0.85	–	[43]
BCFZY^k^	BZCYYb1711	Ni-BZCYYbRF^l^	–	0.45	0.65	0.81	–	[15]
BCFZY	BZCYYbPd^m^	Ni-BZCYYbPd	–	0.37	0.51	0.72	–	[16]
BCFZY	BZCYYb1711	Ni-BZCYYb-KMg^n^	–	–	0.45	–	–	[44]
BCFZY	BZCYYb1711	Ni-BZCYYb1711	BZCYYbNRu^o^ filler	0.12	0.22	0.35	0.48	[45]
PBSCF	BZCYYb1711	Ni_97_Co_3_-BZCYYb1711	–	–	0.48	0.60	0.85	[46]
PBSCF	BZCYYb1711	Fe-decorated Ni-BZCYYb1711	–	0.35	0.71	1.25	1.61	[9]

^a^Ba_0.95_(Co_0.4_Fe_0.4_Zr_0.1_Y_0.1_)_0.95_Ni_0.05_O_3-δ_;

^b^ BaZr_0.1_Ce_0.7_Y_0.1_Yb_0.1_O_3-δ_;

^c^ Pr_0.6_Sr_0.4_(Co_0.2_Fe_0.8_)_0.85_Ru_0.15_O_3-δ_;

^d^ PrBa_0.5_Sr_0.5_Co_1.5_Fe_0.5_O_5+δ_;

^e^ Sr_2_Fe_1.35_Mo_0.45_Cu_0.2_O_6-δ_;

^f^ Cs_2_O-modified Ru catalyst (RC) on the anode prepared by a one-step precursor mixing (OPM-Anode);

^g^ BaCo_0.7_Ce_0.24_Y_0.06_O_3-δ_;

^h^ Ba(Zr_0.1_Ce_0.7_Y_0.1_Yb_0.1_)_0.95_Ni_0.05_O_3-δ_;

^i^ Ru_0.95_Cu_0.05_-Ni_x_;

^j^BaZr_0.2_Ce_0.6_Y_0.1_Yb_0.1_O_3-δ_;

^k^ BaCo_0.4_Fe_0.4_Zr_0.1_Y_0.1_O_3-δ_;

^l^Ba(Zr_0.1_Ce_0.7_Y_0.1_Yb_0.1_)_0.94_Ru_0.03_Fe_0.03_O_3-δ_;

^m^ Ba(Zr_0.1_Ce_0.7_Y_0.1_Yb_0.1_)_0.95_Pd_0.05_O_3−δ_;

^n^Ba_0.95_K_0.05_(Zr_0.1_Ce_0.7_Y_0.1_Yb_0.1_)_0.95_Mg_0.05_O_3-δ_;

^o^ B(Zr_0.1_Ce_0.7_Y_0.1_Yb_0.1_)_0.9_Ni_0.05_Ru_0.05_O_3-δ_

The durability of both cells was further examined under constant current operation of 0.3 A cm^−2^ at 600 °C with NH_3_ fuel. As shown in Fig. 5g, the bare cell exhibited rapid performance decay, attributable to accelerated Ni nitridation and particle coarsening arising from direct exposure to NH_3_. These degradation mechanisms reduce the number of electrochemically active sites and hinder mass transport. In sharp contrast, the SFMMCCN cell maintained stable output for over 255 h of continuous operation without signs of abrupt degradation. Figures 5h, S28, and S29 reveal that while the SFMMCCN cell experienced only a slight increase in non-ohmic resistance up to 200 h before stabilizing, the bare cell exhibited a continuous rise, reflecting ongoing degradation. Post-test SEM analysis (Fig. S30) confirmed that Ni particles in the SFMMCCN cell remained significantly finer than those in the bare cell, reinforcing the protective role of the ACL in mitigating Ni agglomeration and nitridation. In addition, Fig. S31 shows that the exsolved nanoparticles on the SFMMCCN surface remain uniformly dispersed without noticeable coalescence even after long-term operation, demonstrating the intrinsic structural stability of the exsolved alloy under operating conditions. Figure 5i, j schematically illustrates the mechanistic differences in NH_3_ decomposition. In the bare Ni-BZCYYb anode (Fig. 5i), NH_3_ adsorbs onto the Ni surfaces and undergoes sequential dehydrogenation. However, the high energy barrier of this pathway limits reaction kinetics and suppresses effective NH_3_ conversion. By contrast, in the SFMMCCN cell (Fig. 5j), in situ exsolved Ni–Fe–Cu alloy nanoparticles, embedded in the high-entropy oxide matrix, provide abundant acid sites and catalytically active medium-entropy alloy interfaces. This synergistic architecture lowers the barriers for NH_3_ adsorption and dehydrogenation, thereby enhancing charge-transfer kinetics while maintaining effective mass transport. Moreover, the ACL minimizes direct NH_3_ exposure to Ni particles, suppressing agglomeration and ensuring long-term stability.

## Conclusion

In this study, we developed a high-entropy perovskite catalyst layer (SFMMCCN) incorporating in situ exsolved Ni–Fe–Cu alloy nanoparticles to address the limitations of conventional anodes in DA-PCFCs. The tailored composition and entropy-stabilized structure endowed the catalyst with superior activity for NH_3_ decomposition and exceptional electrochemical stability. Compared with the bare cell, the SFMMCCN-based cell achieved a remarkable power output of 2.04 W cm^−2^ at 700 °C and preserved both structural and functional integrity during prolonged operation under NH_3_ at 600 °C. By integrating comprehensive experimental characterization with DFT calculations, we revealed that the enhanced performance originates from favorable exsolution thermodynamics and synergistic alloy-high-entropy oxide interfaces that accelerate reaction kinetics. These findings demonstrate that catalytic efficiency in DA-PCFCs can be substantially advanced through high-entropy materials design. More broadly, this work establishes a rational framework for engineering anode catalyst layers, offering a promising route toward efficient, durable, and scalable NH_3_-to-power technologies.

## Supplementary Information

Below is the link to the electronic supplementary material.

Supplementary file1 (DOCX 9451 kb)
