# Supplementary material for: Entropy-Modulated Oxide–Metal Catalyst Architectures for Direct Ammonia Protonic Ceramic Fuel Cells
Source: Nanomicro Lett. 2026 Apr 17;18:335. doi: 10.1007/s40820-026-02194-9 (PMC13087071; doi:10.1007/s40820-026-02194-9)
Supplement: Supplementary file 1 — Supplementary file1 (DOCX 9451 kb) [file 40820_2026_2194_MOESM1_ESM.docx]

Supplementary Information for

**Entropy-Modulated Oxide–Metal Catalyst Architectures for Direct Ammonia Protonic Ceramic Fuel Cells**

Dongyeon Kim^1,†^, Dong Jae Park^2,†^, Incheol Jeong^3,†^, Seeun Oh^4^, Hyeonggeun Kim^4^, Mincheol Lee^4^, Sang Won Lee^2^, Kangyong Lee^4^, Daehan Chung^4^, Ki-Min Roh^3*^, Joongmyeon Bae^4*^, Tae Ho Shin^2*^, Kang Taek Lee^1,4,5*^

^1^KAIST InnoCORE PRISM-AI Center, KAIST, Daejeon, Republic of Korea

^2^Hydrogen Energy Materials Center, Korea Institute of Ceramic Engineering and Technology (KICET), Jinju-Si, Gyeongsangnam-do, Republic of Korea

^3^Resources Utilization Research Center, Korea Institute of Geoscience and Mineral Resources (KIGAM), Daejeon, Republic of Korea

^4^Department of Mechanical Engineering, KAIST, Daejeon, Republic of Korea

^5^KAIST Graduate School of Green Growth & Sustainability, Daejeon, Republic of Korea

^†^Dongyeon Kim, Dong Jae Park, and Incheol Jeong contributed equally to this work.

* Corresponding authors. E-mail: [kmroh@kigam.re.kr](mailto:kmroh@kigam.re.kr) (Ki-Min Roh); [jmbae@kaist.ac.kr](mailto:jmbae@kaist.ac.kr) (Joongmyeon Bae); [ths@kicet.re.kr](mailto:ths@kicet.re.kr) (Tae Ho Shin); [leekt@kaist.ac.kr](mailto:leekt@kaist.ac.kr) (Kang Taek Lee)

**Supplementary Table and Figues**

**Table S1** Fitted Fe 2p XPS parameters for SFMMCCN in the pristine and reduced conditions


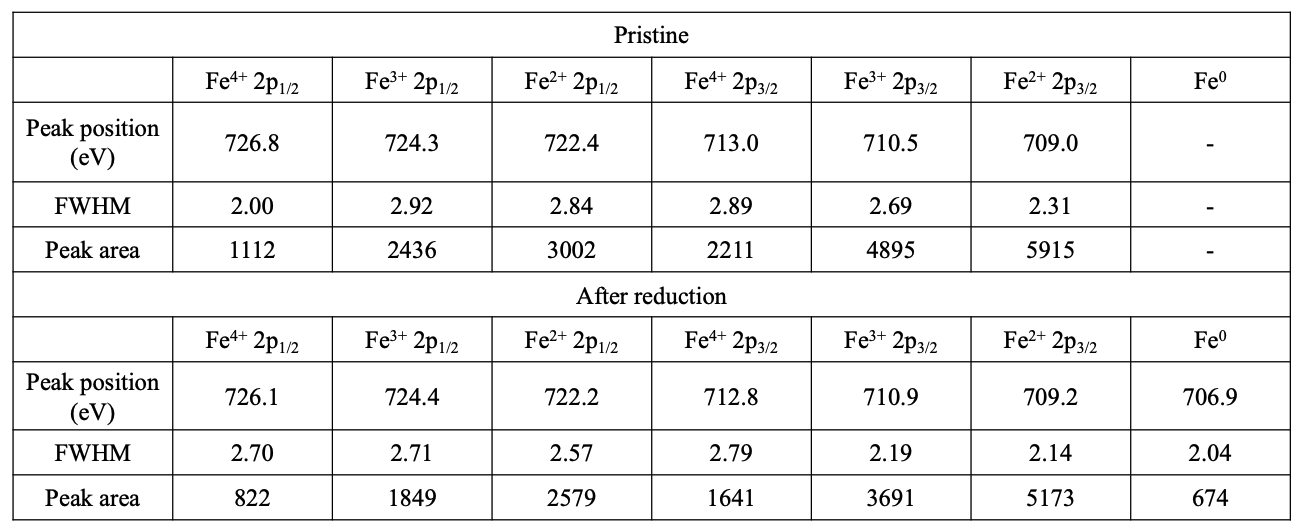


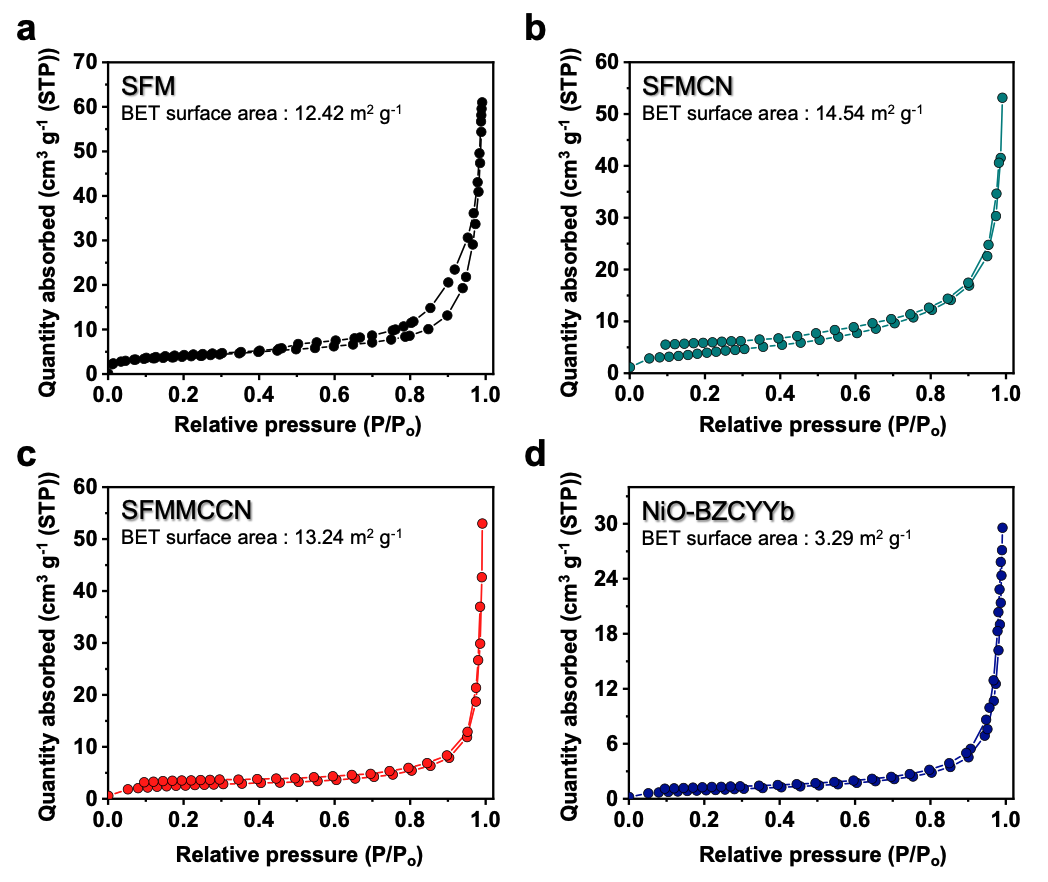


**Fig. S1** BET specific surface areas of SFM, SFMCN, SFMMCCN, and NiO–BZCYYb


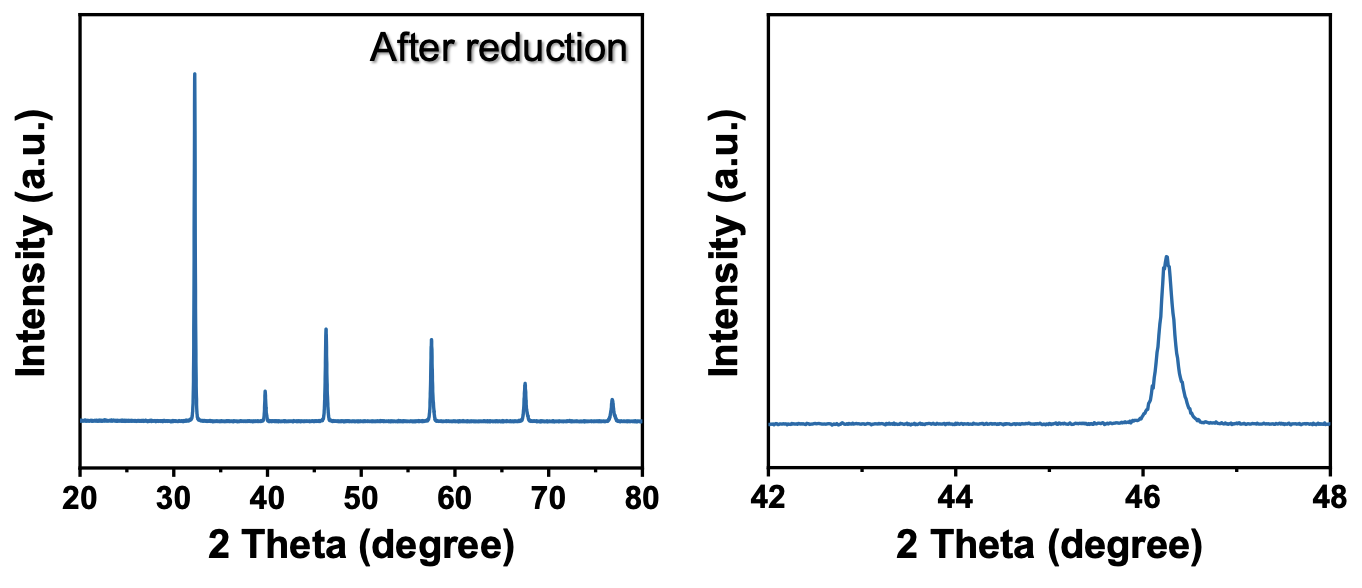


**Fig. S2** XRD patterns of the SFM after heat treatment under a reducing atmosphere (100% H_2_) at 700 ℃


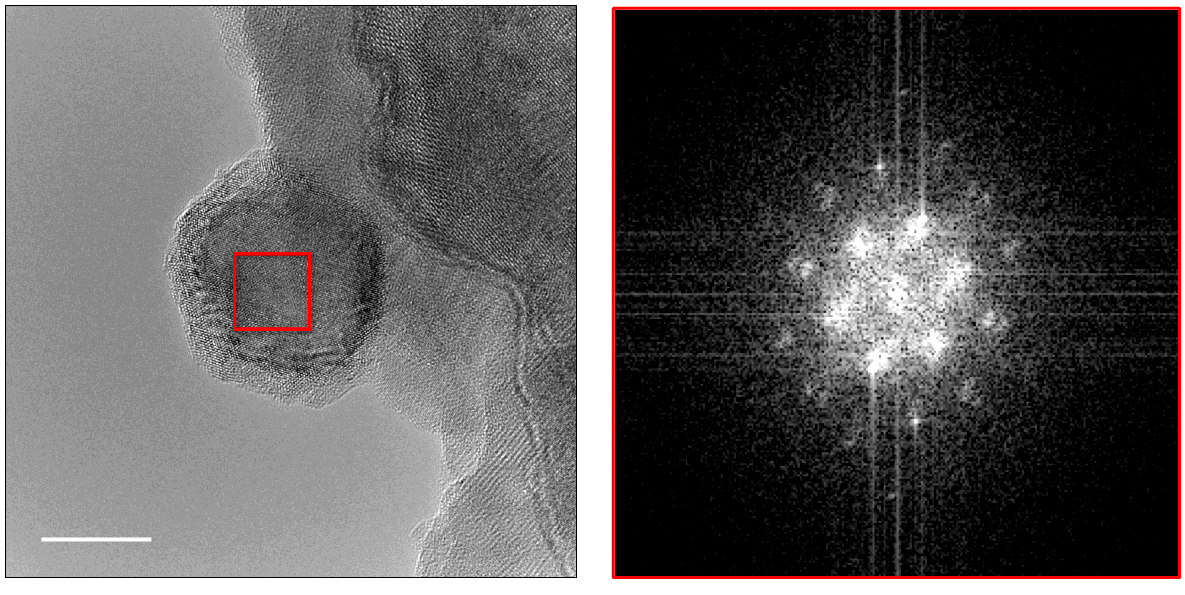


**Fig. S3** HR-TEM image and fast fourier transform(FFT) pattern of exsolved nanoparticle


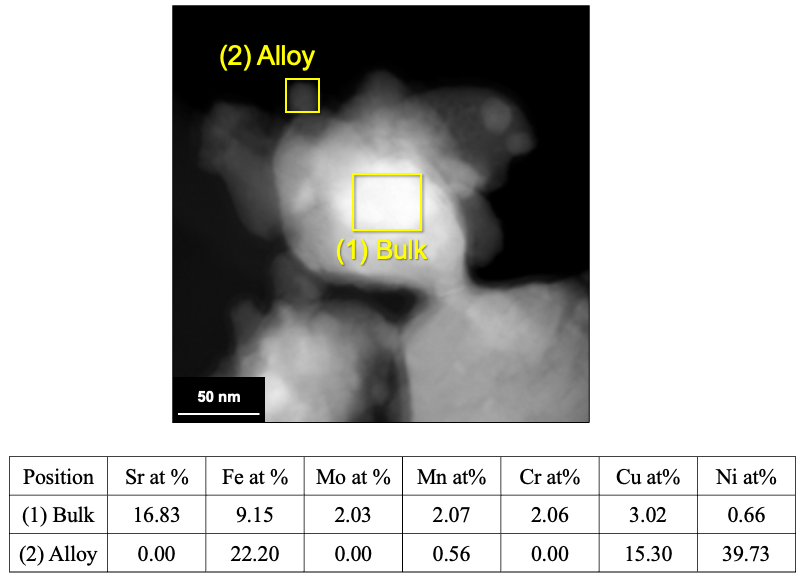


**Fig. S4** STEM-EDS area analyses of the reduced SFMMCCN


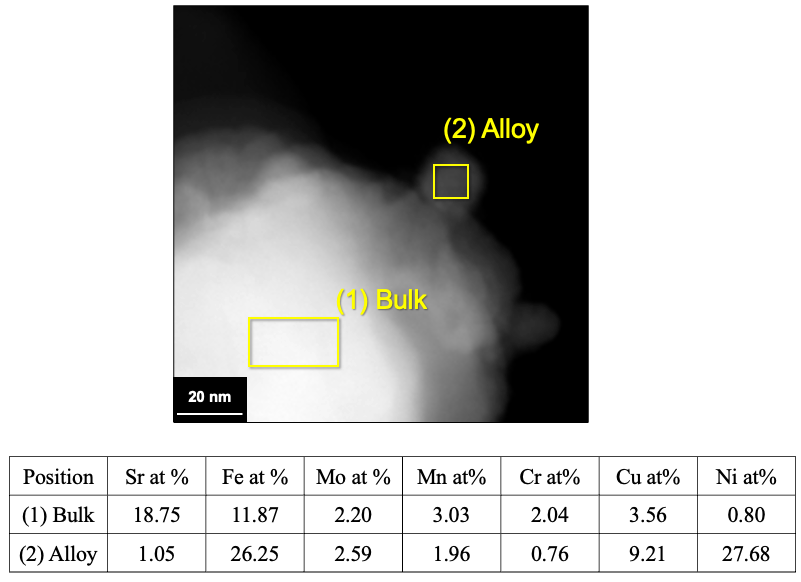


**Fig. S5** STEM-EDS area analyses of the reduced SFMMCCN


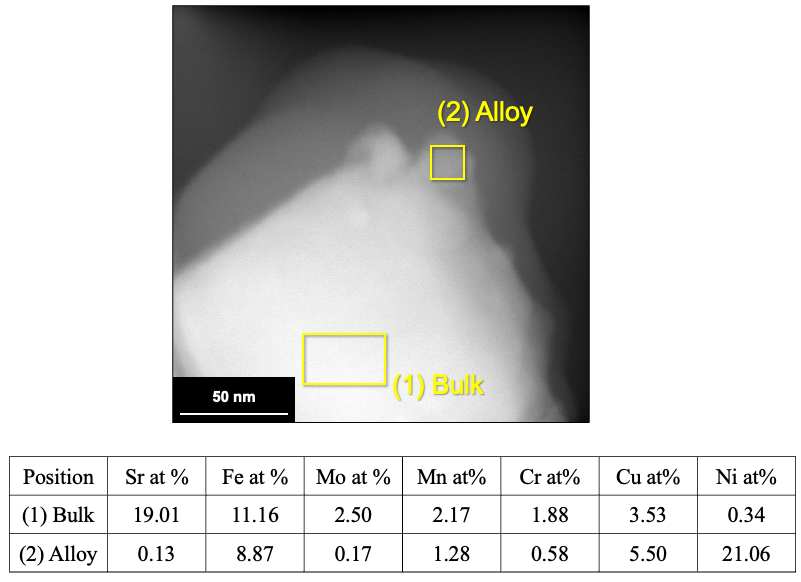


**Fig. S6** STEM-EDS area analyses of the reduced SFMMCCN


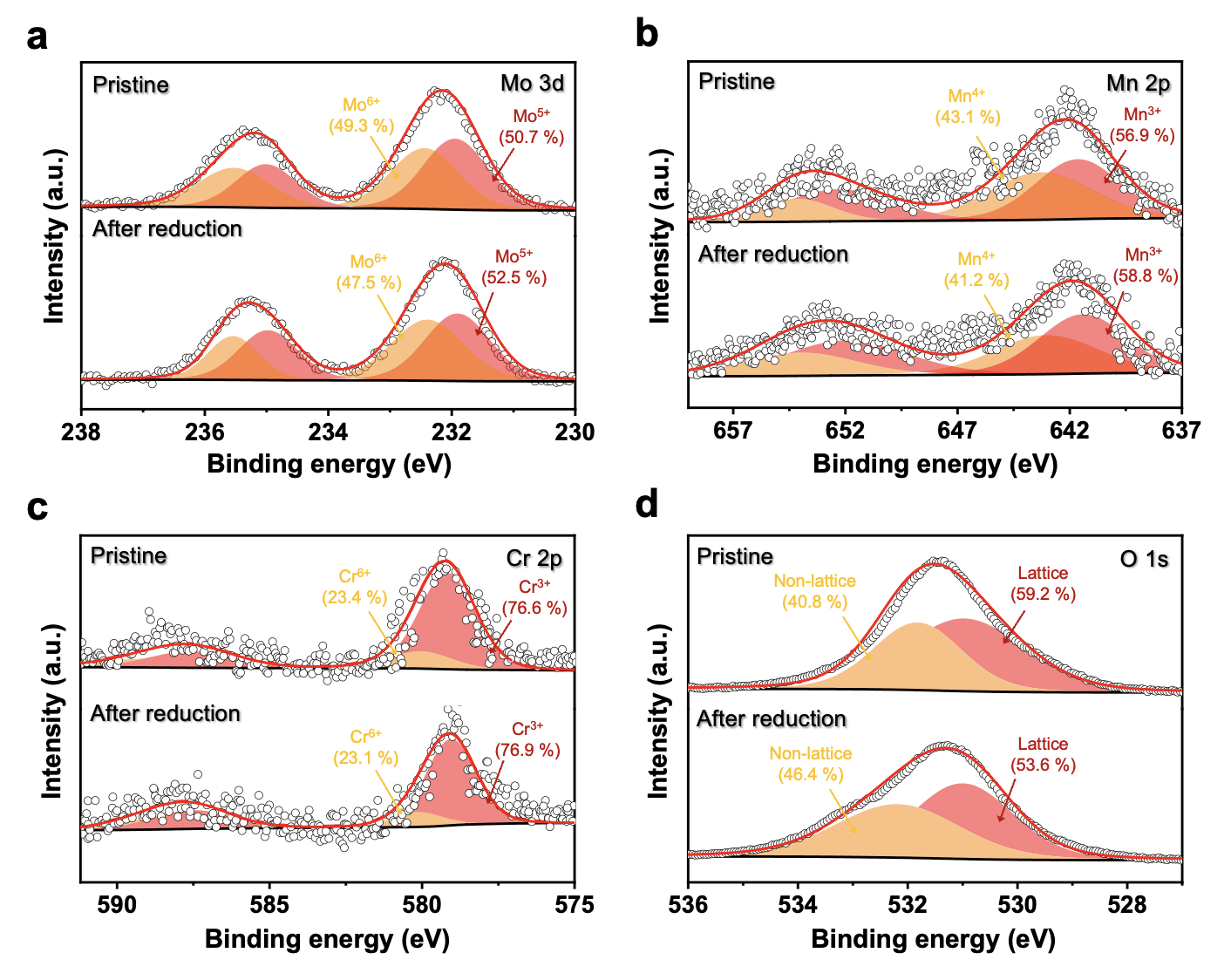


**Fig. S7** XPS spectra of (**a**) Mo 3d, (**b**) Mn 2p, (**c**) Cr 2p, and (**d**) O 1s for SFMMCCN before and after reduction at 700 ℃ for 2h in a 100 % H_2_ atmosphere


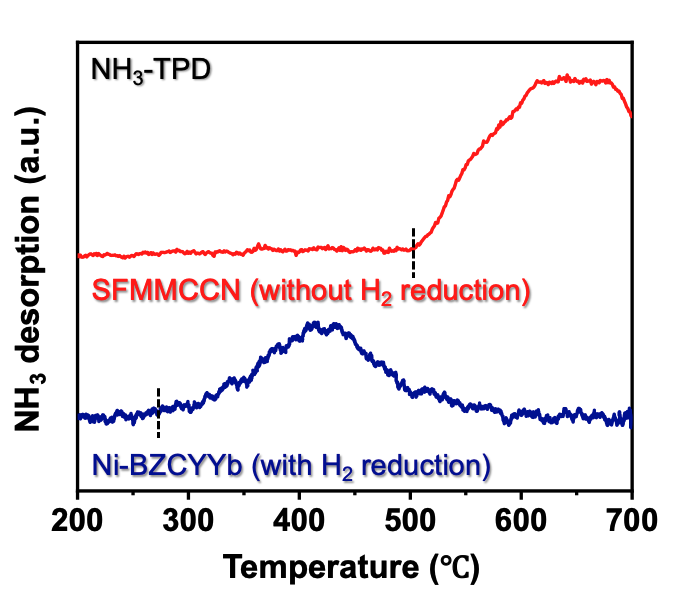


**Fig. S8** NH_3_-TPD profiles of Ni–BZCYYb (with prior H_2_ reduction treatment) and SFMMCCN (without prior H_2_ reduction treatment)


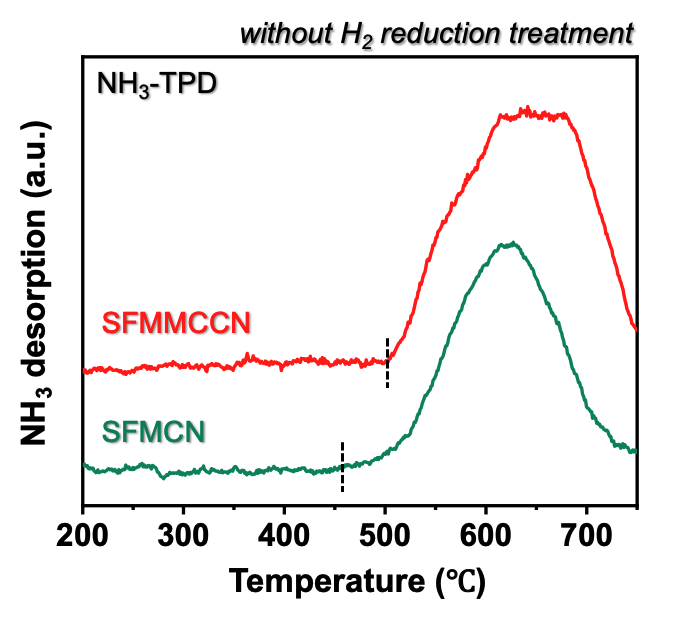


**Fig. S9** NH_3_-TPD profiles of SFMCN and SFMMCCN without prior H_2_ reduction treatment
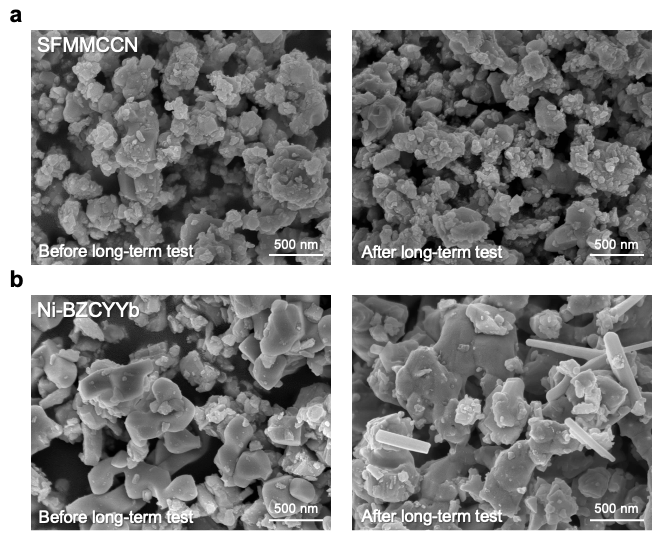


**Fig. S10** SEM images of (**a**) SFMMCCN and (**b**) Ni-BZCYYb powders, obtained before and after long-term NH_3_ conversion test


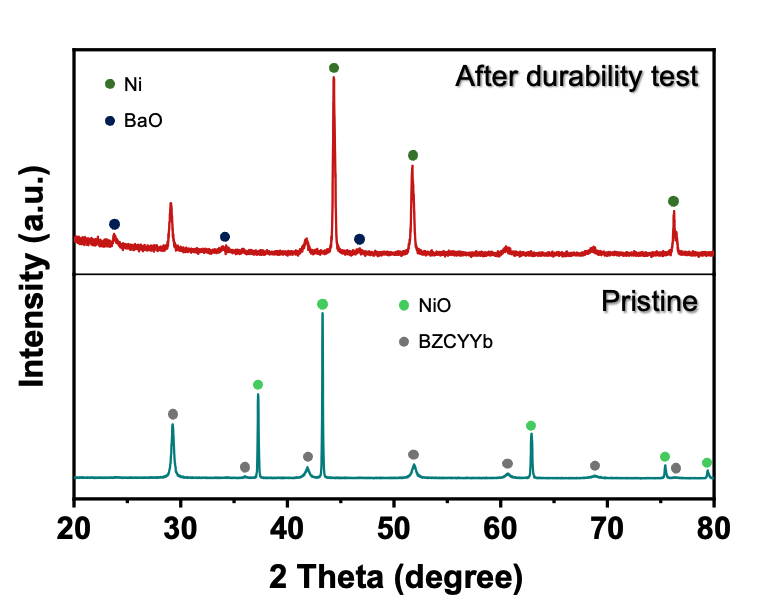


**Fig. S11** XRD patterns of pristine Ni-BZCYYb and Ni-BZCYYb after the durability test under NH_3_ gas conditions


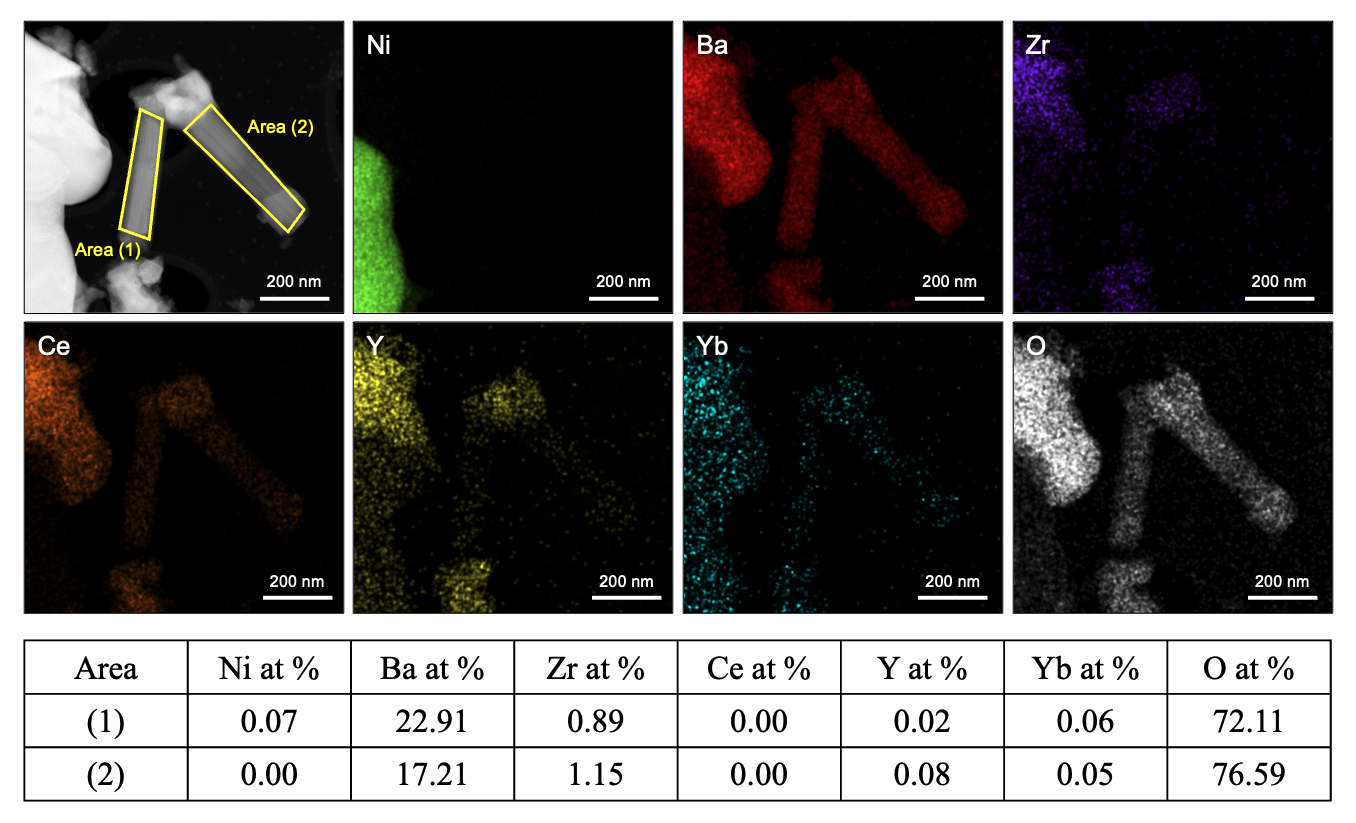


**Fig. S12** XRD patterns of pristine Ni-BZCYYb and Ni-BZCYYb after the durability test under NH_3_ gas conditions


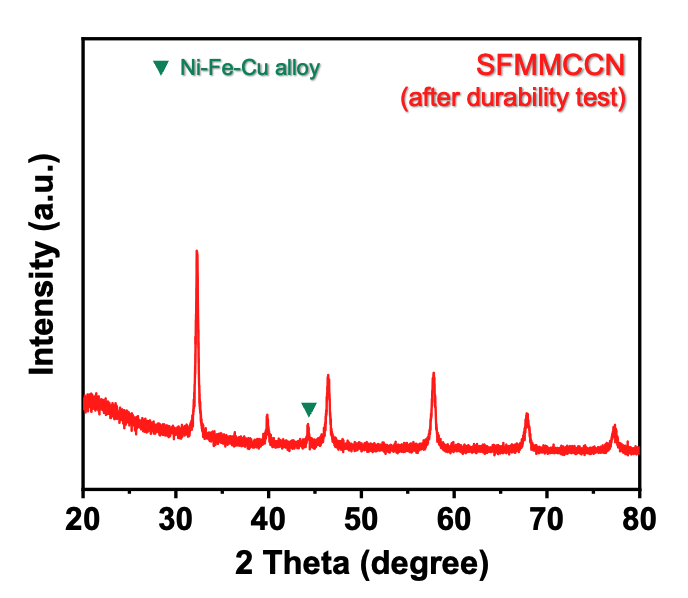


**Fig. S13** XRD pattern of the SFMMCCN catalyst after the durability test under NH_3_ gas conditions


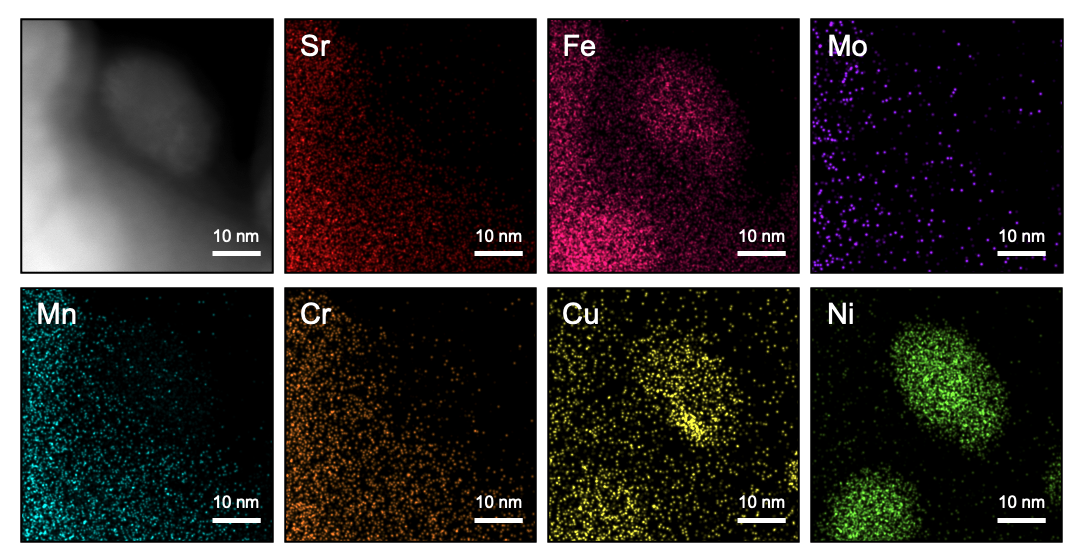


**Fig. S14** STEM-EDS mapping images of the SFMMCCN after the durability test under NH_3_ gas conditions


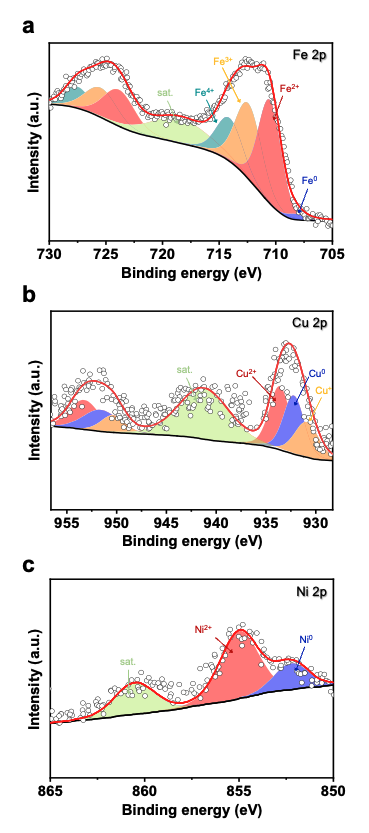


**Fig. S15** STEM-EDS mapping images of the SFMMCCN after the durability test under NH_3_ gas conditions


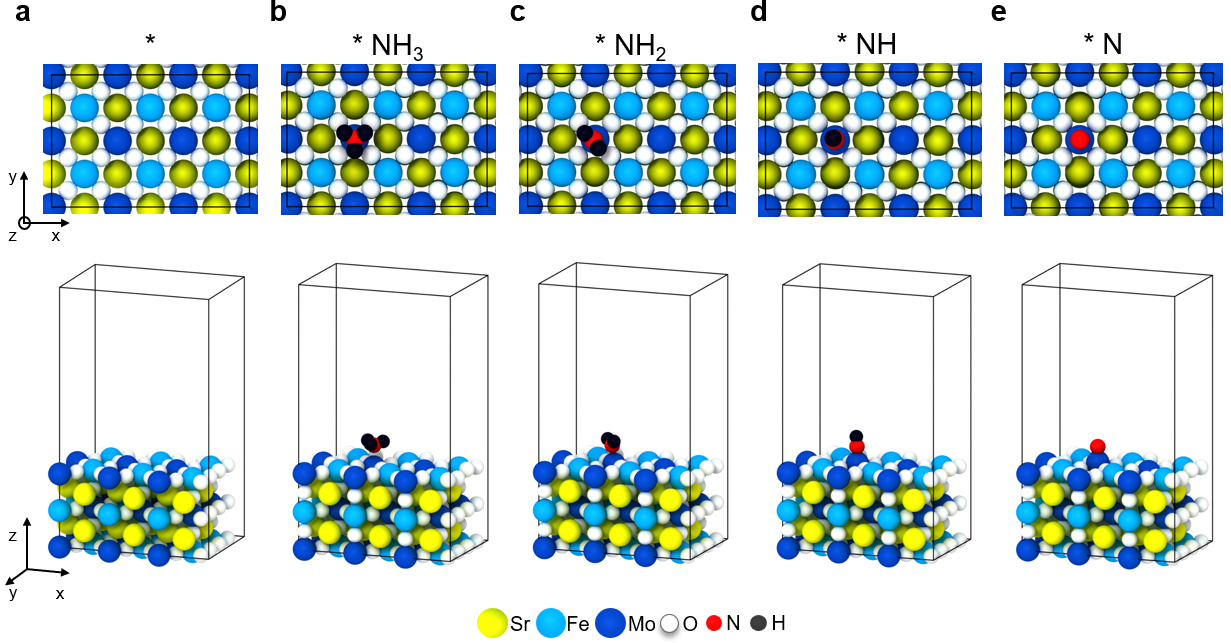


**Fig. S16** Optimized SFM slab structure models including (**a**) pristine slab, slabs with adsorbed (**b**) NH_3_, (**c**) NH_2_, (**d**) NH, and (**e**) N species


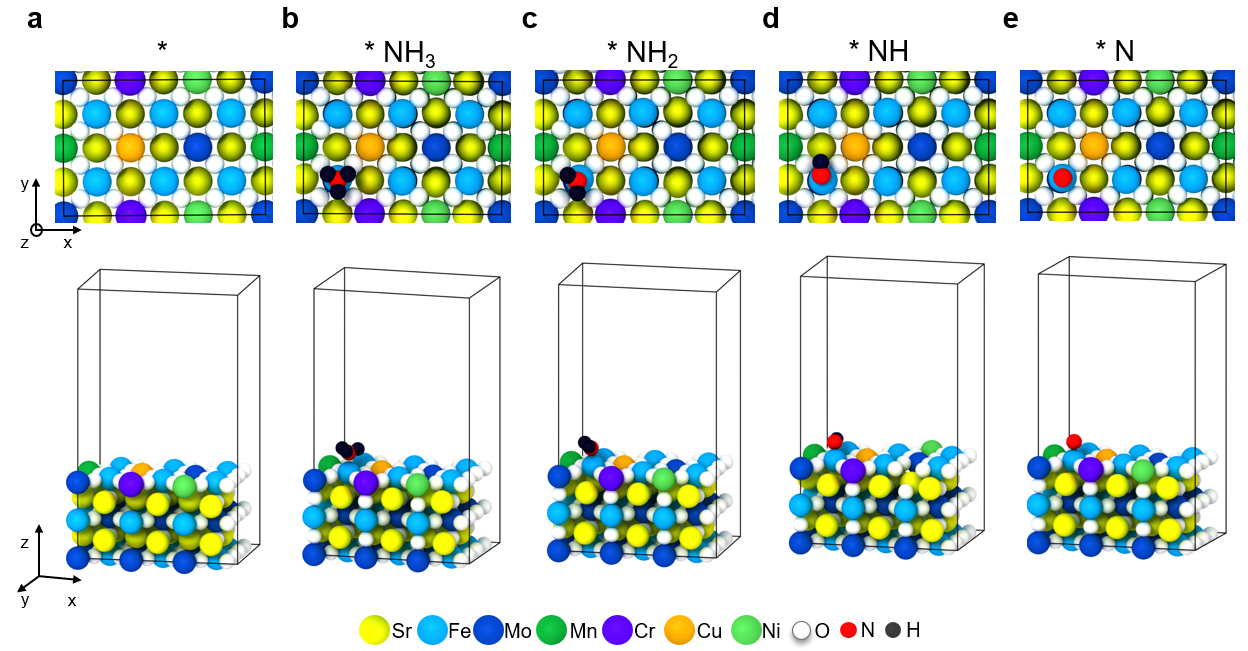


**Fig. S17** Optimized SFMMCCN slab structure models including (**a**) pristine slab, slabs with adsorbed (**b**) NH_3_, (**c**) NH_2_, (**d**) NH, and (**e**) N species


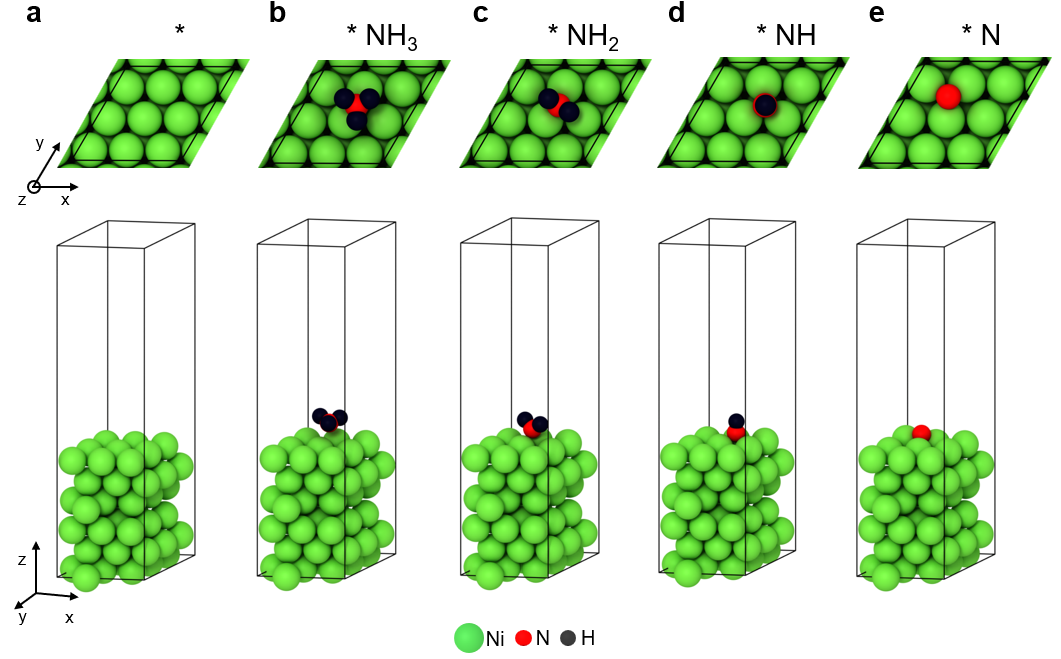


**Fig. S18** Optimized SFMMCCN slab structure models including (**a**) pristine slab, slabs with adsorbed (**b**) NH_3_, (**c**) NH_2_, (**d**) NH, and (**e**) N species


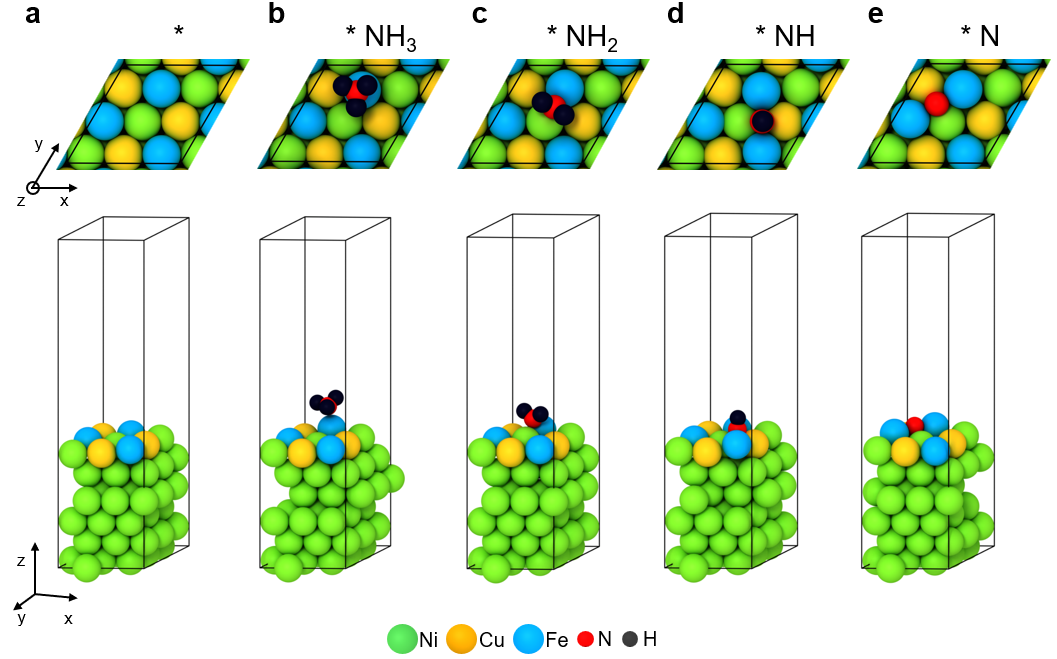


**Fig. S19** Optimized Ni-Fe-Cu slab structure models including (**a**) pristine slab, slabs with adsorbed (**b**) NH_3_, (**c**) NH_2_, (**d**) NH, and (**e**) N species


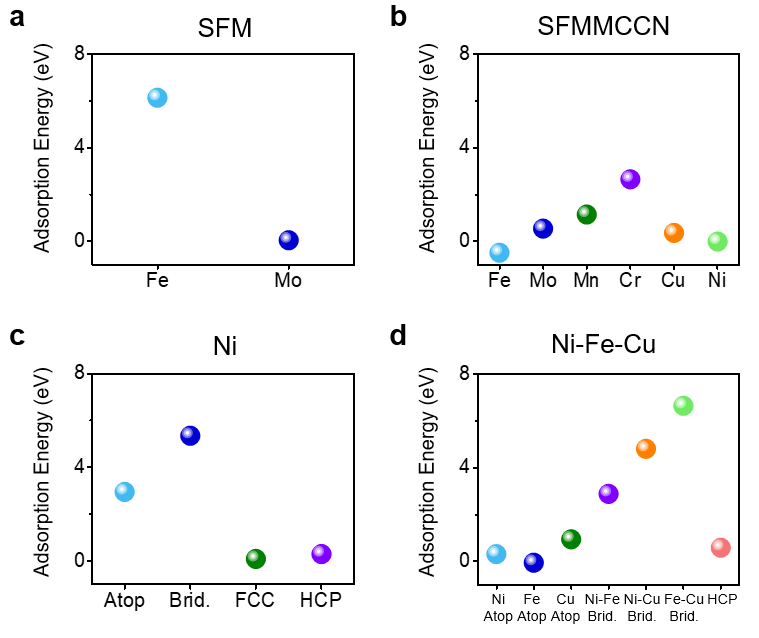


**Fig. S20** Optimized Ni-Fe-Cu slab structure models including (**a**) pristine slab, slabs with adsorbed (**b**) NH_3_, (**c**) NH_2_, (**d**) NH, and (**e**) N species


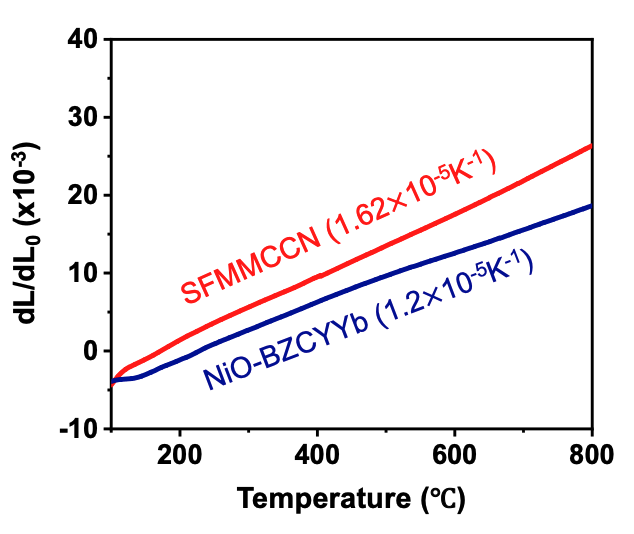


**Fig. S21** Optimized Ni-Fe-Cu slab structure models including (**a**) pristine slab, slabs with adsorbed (**b**) NH_3_, (**c**) NH_2_, (**d**) NH, and (**e**) N species


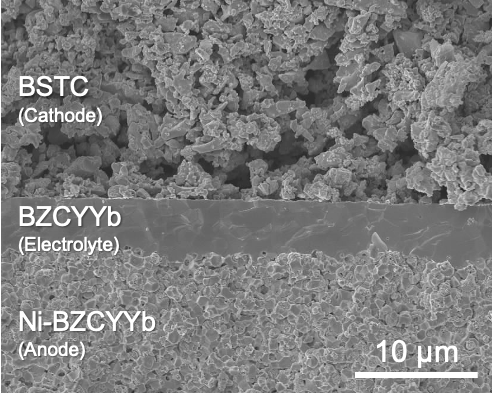


**Fig. S22** Cross-sectional SEM image of bare cell


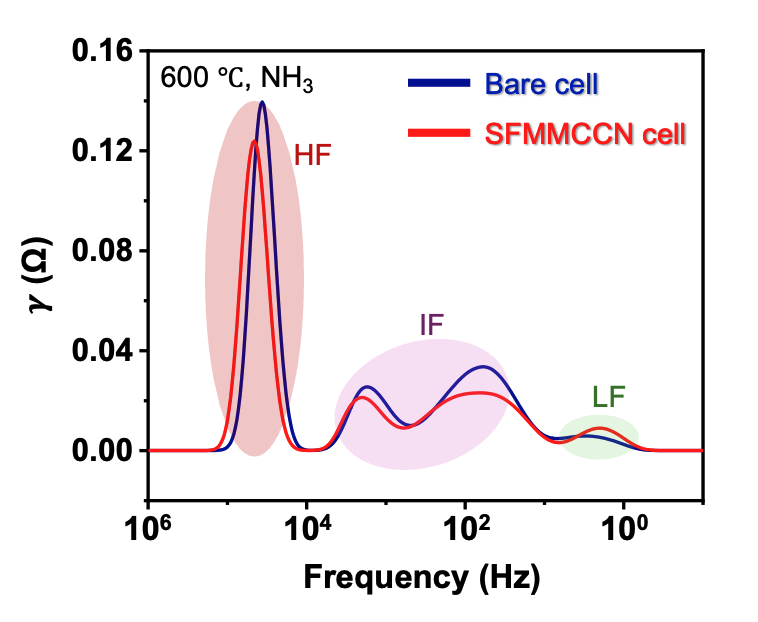


**Fig. S23** Distribution of relaxation time (DRT) plots of the bare cell and the SFMMCCN cell at 600 ℃ under NH_3_ gas conditions


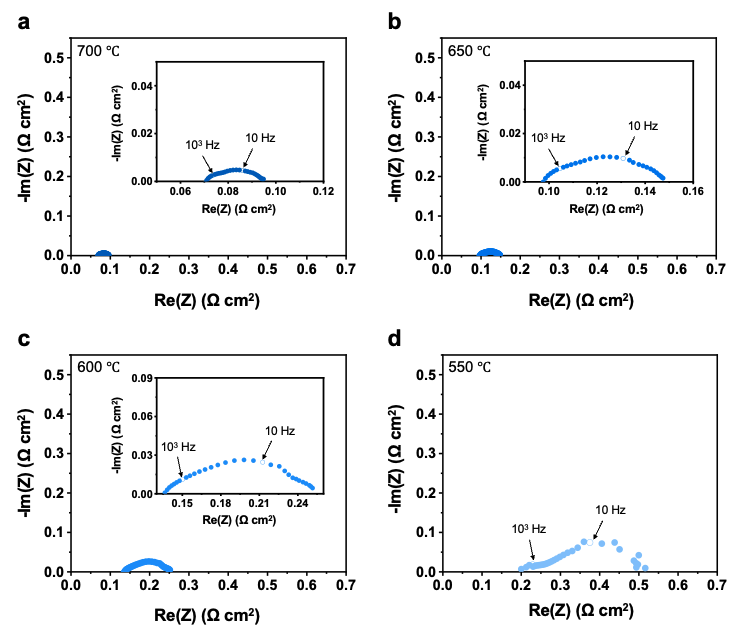


**Fig. S24** Nyquist plots of the SFMMCCN cell measured at temperatures of (**a**) 700, (**b**) 650, (**c**) 600, and (**d**) 550 ℃


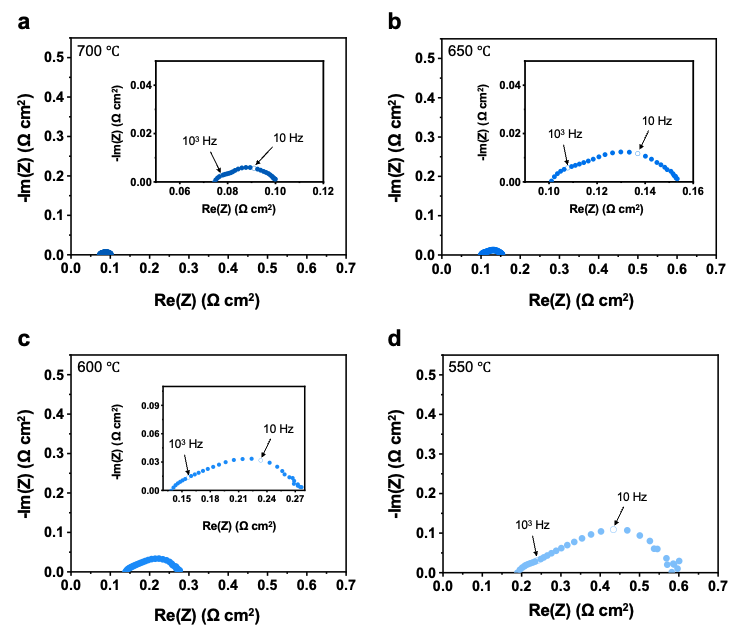


**Fig. S25** Nyquist plots of the bare cell measured at temperatures of (**a**) 700, (**b**) 650, (**c**) 600, and (**d**) 550 ℃


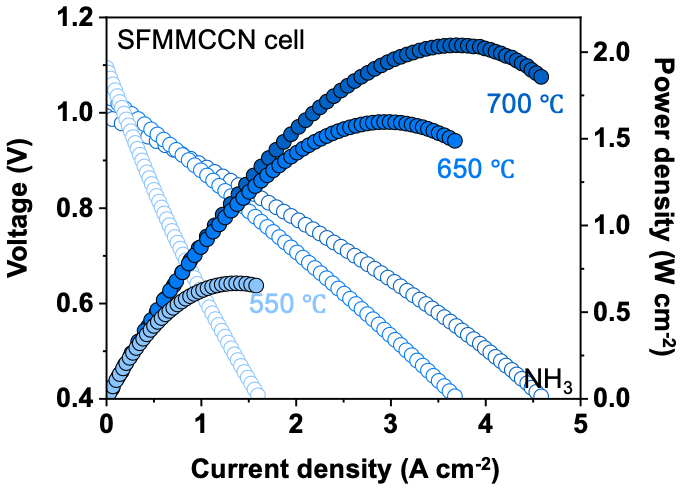


**Fig. S26** *I-V-P* curves of the SFMMCCN cell measured over a temperature range from 700 to 550 ℃


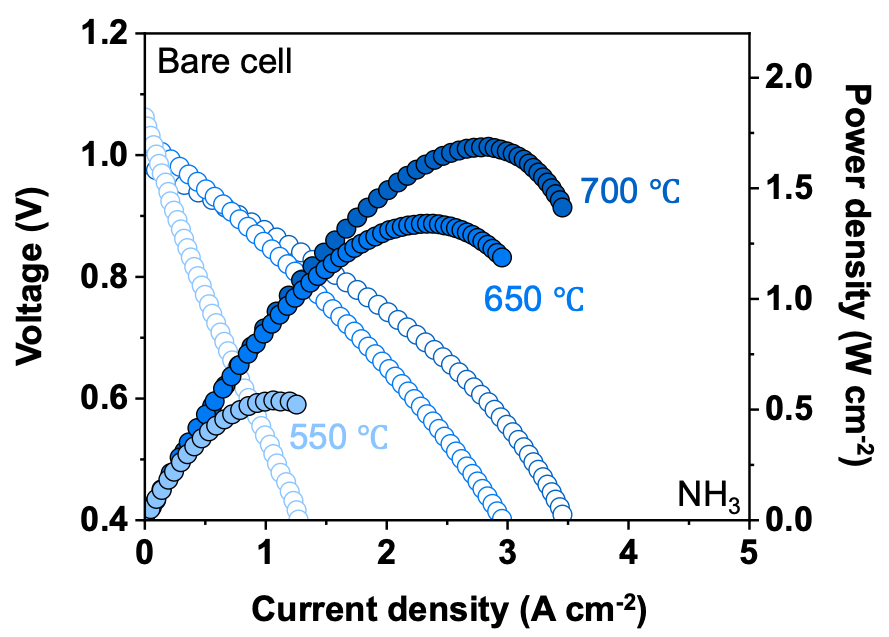


**Fig. S27** *I-V-P* curves of the bare cell measured over a temperature range from 700 to 550 ℃

**Fig. S28** Nyquist plot of the SFMMCCN cell measured at various time intervals during the long-term durability test

**Fig. S29** Nyquist plot of the bare cell measured at various time intervals during the long-term durability test


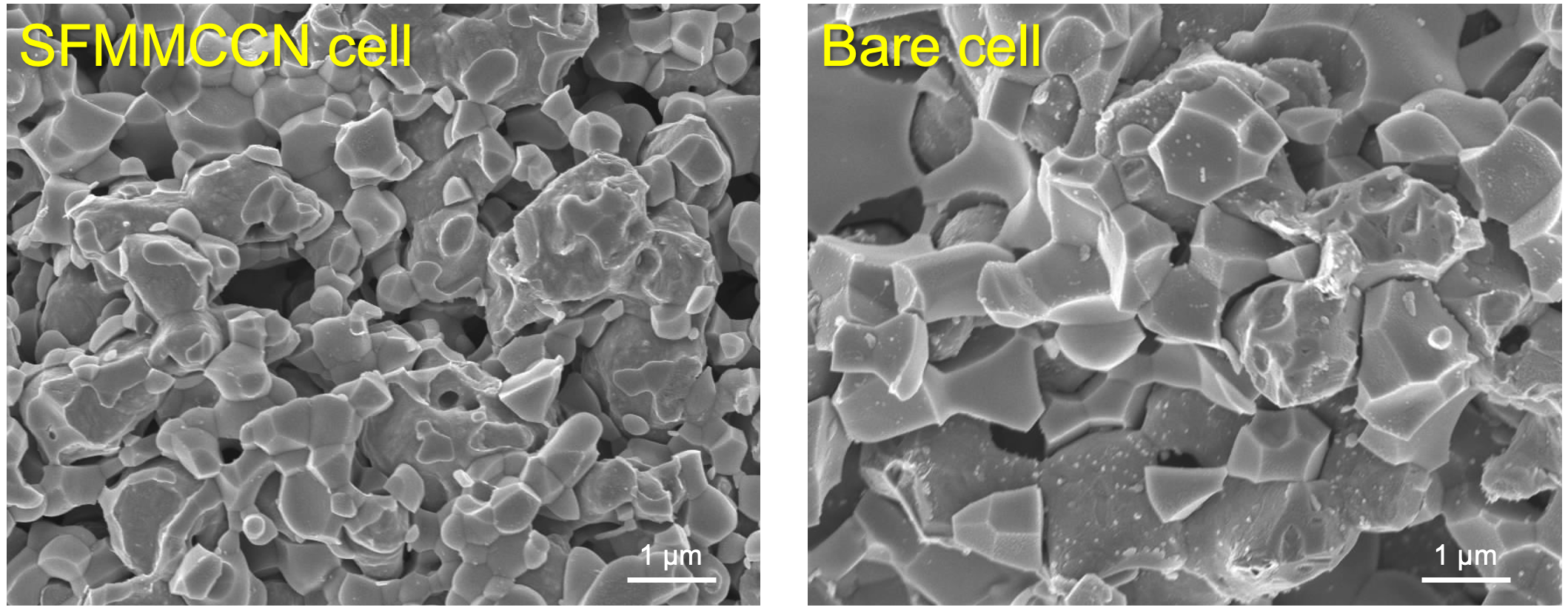


**Fig. S30** SEM images of the anode in SFMMCCN cell and bare cell, obtained after long-term stability test under NH_3_ fuel


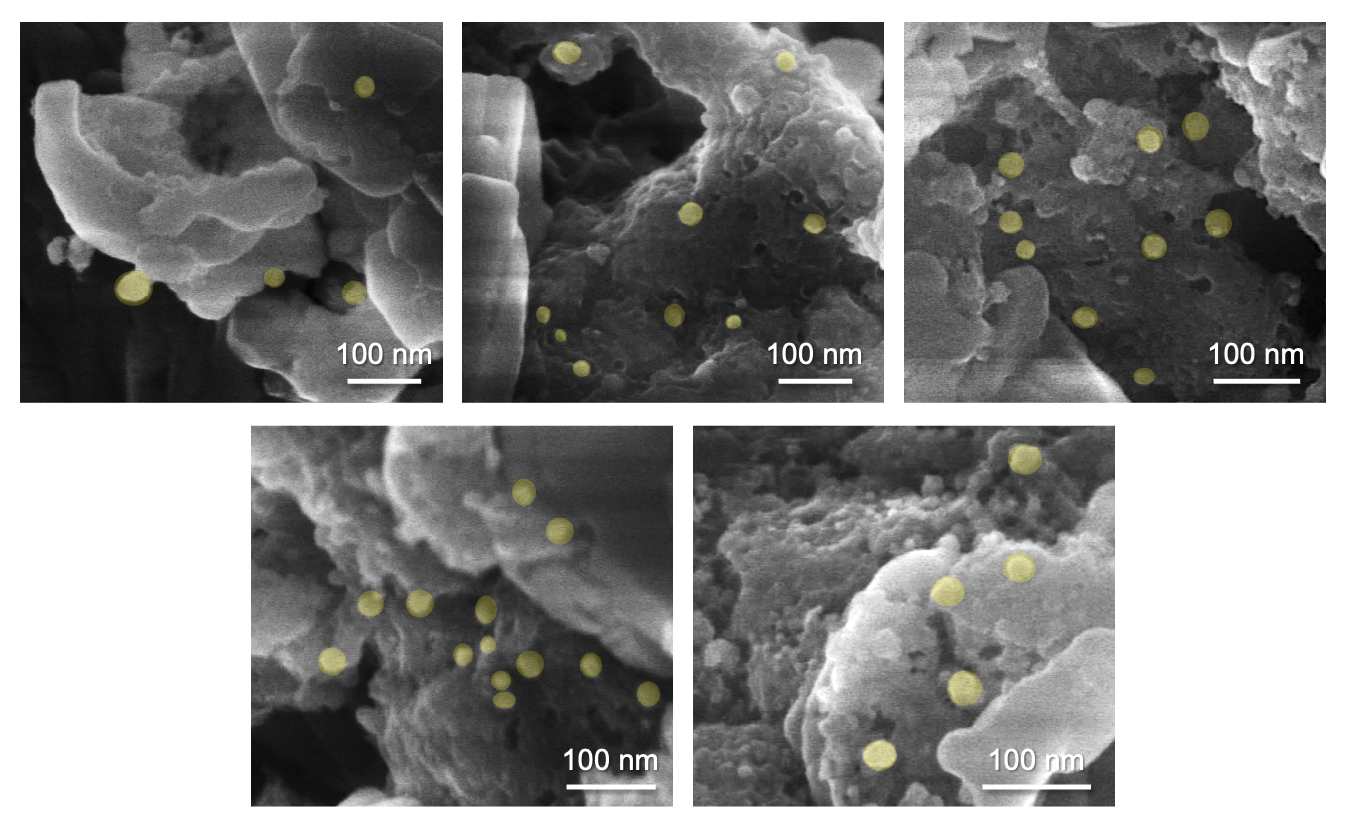


**Fig. S31** SEM images of the SFMMCCN ACL after the durability test at 600 ℃
